# Detection of psychosis risk: reliability and validity of the Spanish version of the Comprehensive Assessment of At-Risk Mental States interview (CAARMS-S)

**DOI:** 10.3389/fpsyg.2026.1726125

**Published:** 2026-04-13

**Authors:** Ana Barajas, Luis Mauri, Jordi Cid, Enrique Gutiérrez, Ana Calvo, Lluis Lalucat

**Affiliations:** 1 Department of Clinical and Health Psychology, Autonomous University of Barcelona, Barcelona, Spain; 2 Serra Húnter Programme, Government of Catalonia, Barcelona, Spain; 3 Sant Pere Claver Fundació Sanitària, Barcelona, Spain; 4 Mental Health & Addiction Research Group, IdiBGi, Institut d’Assistència Sanitària, Girona, Spain; 5 Department of Applied Mathematics for Information and Communication Technologies, Higher Technical School of Computer Systems Engineering (NEBULA Research Group), Madrid, Spain; 6 MIT linQ - Massachusetts Institute of Technology, Cambridge, MA, United States; 7 Department of Personality, Assessment and Clinical Psychology, School of Psychology, Universidad Complutense de Madrid, Madrid, Spain; 8 Department of Research, Centre d’Higiene Mental Les Corts, Barcelona, Spain

**Keywords:** at-risk, CAARMS, early detection, first-episode psychosis, psychosis, ultra-high risk, validation

## Abstract

**Introduction:**

The early identification of psychosis risk is critical, particularly within the first 2 years of the at-risk phase, as timely intervention may reduce the likelihood of transitioning to psychosis. This study evaluates the reliability and validity of the Spanish version of the Comprehensive Assessment of At-Risk Mental States (CAARMS-S) interview.

**Methods:**

A prospective longitudinal study was conducted with 36 individuals at ultra-high risk (UHR), 43 with First-Episode Psychosis (FEP), and 14 matched controls, all of whom were part of the Early Intervention Program in Catalonia. Data collection involved clinical interviews and multiple validated scales, including the CAARMS-S, the Positive and Negative Syndrome Scale (PANSS), the Global Assessment of Functioning (GAF), the Structured Clinical Interview for DSM-IV Axis II Personality Disorders (SCID-II), and the Kiddie-Schedule for Affective Disorders and Schizophrenia (K-SADS-PL).

**Results:**

Reliability testing showed excellent internal consistency (Cronbach’s *α* = 0.93) and inter-observer reliability [intraclass correlation coefficient (ICC) = 0.92]. Construct validity was supported by significant differences in PANSS positive subscale scores among UHR, FEP, and control groups (*p* < 0.001). Strong correlations were observed between CAARMS-S and PANSS subscales (positive: *r* = 0.608; negative: *r* = 0.495; general: *r* = 0.577). After 6 months, only one UHR participant (3.3%) transitioned to psychosis. Both CAARMS-S and PANSS were sensitive to changes in positive symptoms, with CAARMS-S being more responsive to negative symptoms and PANSS to general psychopathology.

**Discussion:**

These findings confirm that CAARMS-S is a reliable, valid, and sensitive tool for identifying and monitoring individuals at risk of psychosis in Spanish-speaking clinical settings, supporting its use in early intervention programs.

## 1 Introduction

Over the past two decades, various early intervention services for psychosis have been developed worldwide. A key requirement for these programs to function effectively is the ability to detect individuals at high risk for psychosis. Early detection in psychosis refers to the correct identification of individuals at risk of developing a psychotic disorder. The identification of psychosis risk has been proposed as a strategy that may: (a) help reduce disability and adverse health and social impacts associated with the at-risk phase; (b) allow earlier clinical support before symptoms and functional impairment become entrenched; and (c) contribute to delaying or ameliorating the onset of a diagnosable psychotic disorder (Yung et al., 2005). However, current evidence indicates that these outcomes have not been consistently demonstrated empirically (Fusar-Poli et al., 2020). Moreover, early detection is essential because the rate of progression to psychosis among individuals at ultra-high risk (UHR) is highest in the first months after presentation. Half of those at UHR who progress to psychosis within 2 years will do so within the first 8 months after presentation to high-risk services (Kempton et al., 2015). In this sense, early identification is key to indicated prevention. However, the boundaries between the asymptomatic phase, the risk phase, and the threshold for frank psychotic symptoms are unclear, and traditional psychopathological assessments are not sufficiently sensitive to subthreshold psychotic symptoms.

In an attempt to overcome these obstacles, different research teams have sought to improve the performance of their instruments for evaluating the risk of psychosis, and a number of new tools have been constructed. Several previous studies (Daneault et al., 2013; Olsen and Rosenbaum, 2006) have provided an overview of existing instruments used for assessing individuals assumed to be at increased risk of psychosis. These studies have considered several approaches to classify these instruments: (a) the attenuated psychotic symptoms (APS) approach, which includes the instruments that pay most attention to the late at-risk phase (1–2 years before the onset of the psychotic episode; Tandon et al., 2012) to identify individuals at risk of imminent conversion to psychosis; (b) the basic symptoms (BS) approach is based on a detailed phenomenological description of subtle, subjectively experienced subclinical disturbances in drive, stress tolerance, affect, thinking, speech, bodily experience, sensory perception, motor activity, and central vegetative functions, which precede the onset of psychosis and are thought to characterize the early at-risk phase, approximately 5 years before the first psychotic episode (Huber and Gross, 1989; Tandon et al., 2012); and (c) the APS-BS approach comprising tools that combine theoretical concepts from both previous approaches (Schultze-Lutter et al., 2016).

Given the observed transition risk of 18% at 6 months, 22% at 1 year, 29% at 2 years, and 36% at 3 years reported in early meta-analytic studies (Fusar-Poli et al., 2012b), a tool is needed to define the late at-risk phase and identify individuals who may transition to psychosis over a 1–2-year period (Cannon et al., 2008; Yung et al., 2003). However, more recent meta-analyses and large-scale longitudinal studies have consistently reported substantially lower transition rates in contemporary UHR cohorts. Current estimates often range between 9 and 11% at 12 months and around 10–22% over longer follow-up periods (Fusar-Poli et al., 2020; Hartmann et al., 2016). This decline has been attributed to earlier detection strategies, sample dilution effects, and the widespread implementation of preventive and needs-based interventions within early psychosis services (Fusar-Poli et al., 2016; van Os and Guloksuz, 2017).

A representative tool of the APS approach is the Comprehensive Assessment of At-Risk Mental States (CAARMS) (Yung et al., 2006), a diagnostic interview and rating system developed by Australian researchers to prospectively assess psychosis risk criteria. It should be noted that the CAARMS is typically administered to help-seeking populations, as these individuals are more likely to present with emerging or attenuated psychotic symptoms, making early detection feasible and clinically relevant. In fact, the CAARMS has two functions: (a) to provide a comprehensive assessment of psychopathology thought to indicate imminent development of a First-Episode Psychosis (FEP) disorder, and (b) to determine if an individual meets UHR status based on criteria derived from the CAARMS assessment (Yung et al., 2005). Accordingly, the CAARMS allows clinicians to classify individuals based on UHR criteria (Yung et al., 2003): (a) APS group, individuals who have experienced subthreshold, attenuated positive psychotic symptoms during the past year; (b) Brief Limited Intermittent Psychotic Symptoms (BLIPS) group, individuals who have symptoms of psychotic intensity, but which are very infrequent, or which have a total duration of less than 7 days before resolving spontaneously; and (c) Vulnerability group, individuals who have a first-degree relative with a psychotic disorder, or who themselves have a schizotypal personality disorder, and who have experienced a significant decrease in functioning during the past year. In addition, the CAARMS identifies individuals who have reached the threshold for frank psychotic symptoms. It has been designed for repeated use over time, for example, from monthly to every 6 months.

The original English early version of the CAARMS demonstrated good reliability and predictive validity (Yung et al., 1996, 2003), and the revised version showed good to excellent concurrent, discriminative, and predictive validity as well as excellent interrater reliability (Yung et al., 2005). Nevertheless, recent meta-analytic evidence suggests that the CAARMS remains a robust and reliable instrument for identifying individuals at risk for psychosis. However, its predictive validity for transition should be considered moderate rather than excellent and is strongly dependent on contextual factors such as clinical setting, duration of follow-up, baseline risk enrichment, and exposure to early intervention treatments (Fusar-Poli et al., 2020; Oliver et al., 2018). To date, the CAARMS has been formally translated and validated in Japanese (Miyakoshi et al., 2009), Italian (Fusar-Poli et al., 2012a; Pelizza et al., 2019), Polish (Jaracz et al., 2012), Arab (Braham et al., 2014), French (Krebs et al., 2014), Greek (Kollias et al., 2015), Korean (Lho et al., 2021), Turkish (Yokuşoğlu et al., 2021) and Pakistani (Jaffar et al., 2025) population.

Regarding the assessment of UHR symptoms in the Spanish population, a precise, culturally and linguistically adapted tool widely used internationally will enable comparability with international studies that use the same instrument. For this reason, we have specifically developed a Spanish version of the CAARMS (CAARMS-S), and analyzed its psychometric properties. The specific aims of this study are the following: (a) to translate and back-translate the CAARMS; (b) to examine the reliability indicators (internal consistency and inter-observer reliability) and validity (construct, concurrent, and predictive validity) of the Spanish version of the CAARMS interview (CAARMS-S). To allow comparison between different validated versions of the CAARMS, we followed the methodological procedure employed by Miyakoshi et al. (2009), Braham et al. (2014), and Pelizza et al. (2019) to validate the Japanese, the Arabic, and the Italian versions of the CAARMS, respectively.

## 2 Methods

### 2.1 Participants

The total sample consisted of 93 participants, including help-seeking individuals at risk for psychosis and healthy volunteers recruited from outpatient psychiatric services located in Catalonia, specifically at one of the following three participating mental health centers: Institut d’Assistència Sanitària (Girona), Fundación Sant Pere Claver (Barcelona), and Asociación Centro de Higiene Mental Les Corts (Barcelona) (the last as a coordinating center).

Patients were enrolled using a consecutive sampling strategy, reflecting a help-seeking clinical population at risk for psychosis. The sample size was determined based on feasibility within the recruitment period, considering the number of participants that could realistically be enrolled from outpatient services during the study timeframe. This approach was guided by previous studies validating the CAARMS interview in similar help-seeking populations assessing psychosis risk (Braham et al., 2014; Fusar-Poli et al., 2012a; Miyakoshi et al., 2009).

Participants were referred from primary outpatient care centers when there was clinical suspicion of emerging psychosis and potential eligibility for an early psychosis program. Subsequently, referring clinicians determined whether individuals met preliminary inclusion criteria, and eligible participants were referred to the study team for assessment. Three groups were formed: (1) First-Episode Psychosis (FEP) group, which included individuals who met the threshold for frank psychotic symptoms according to an adapted version of the Brief Psychiatric Rating Scale (BPRS) / Comprehensive Assessment of Symptoms and History (CASH) criteria (Yung et al., 2005) using the PANSS scale (see Supplementary S1). Exclusion criteria were: (a) a past episode of frank psychosis consistent with affective or non-affective psychotic disorders according to *Mental and Behavioral Disorders Section of the International Classification of Disorders and Related Health Problems, Tenth Revision*, (ICD-10) criteria; (b) known intellectual disability [Intelligence Quotient (IQ) < 70] and (c) somatic pathology (assessed through the medical history) with mental repercussions and/or loss of consciousness; (2) UHR group, which included individuals fulfilling UHR criteria according to an adapted version of the BPRS/CASH criteria (Yung et al., 2005) using PANSS scale (see Supplementary S2). Inclusion requires one or more of the following: APS, BLIPS, or a trait vulnerability plus a marked decline in psychosocial functioning (Genetic Risk and Deterioration Syndrome: GRDS). Exclusion criteria matched those for the FEP group, with the additional exclusion of a current psychotic episode; (3) Control group, which included healthy volunteers matched with FEP and UHR groups by sociodemographic variables (sex, age, and educational level).

All participants were aged between 14 and 35 years, had adequate Spanish comprehension, and provided signed informed consent. The project was evaluated by the research and ethics committees of the coordinating center and each center included in the study.

### 2.2 Measures

*Sociodemographic and clinical characteristics*. The sociodemographic characteristics (date of birth, age, gender, level of education, occupational status, current marital status, and current living situation) and clinical features of the sample [duration untreated illness (DUI), age at onset of psychotic symptoms, prescription of antipsychotic treatment, diagnosis and family history] were obtained with a questionnaire created specifically for this study. Other information was obtained from the following set of psychometric instruments:

*Diagnosis*. To assess compliance with the inclusion and exclusion criteria, diagnosis was established, depending on patient age, through the following instruments: the Structured Clinical Interview for DSM-IV Axis I (SCID-I) (First et al., 1996) for adults and the Kiddie-Schedule for Affective Disorders and Schizophrenia (K-SADS-PL) (Kaufman et al., 1997; Ulloa et al., 2006) for adolescents. The K-SADS-PL was administered separately to parents and patients by trained researchers. Schizotypal personality disorder was determined using the Structured Clinical Interview for DSM-IV Axis II (SCID-II) (First et al., 1997).

*Psychopathological measures*. The CAARMS (Yung et al., 2005) is a semi-structured interview administered to assess attenuated and threshold psychotic symptoms. It is used to determine whether a person meets the UHR criteria and to assess transition to psychosis. The CAARMS manual (Yung and O’Dwyer, 2006) provides detailed definitions, questions, and anchor points for eliciting and rating 28 symptoms (items) across seven dimensions of psychopathology, including positive symptoms, cognitive change, emotional disturbance, negative symptoms, behavioral change, motor physical change, and general psychopathology. Only scores on the positive symptoms subscales are included when evaluating UHR criteria. Dimensions of intensity (ranging from 0 to 6) and frequency/duration (ranging from 0 to 6) are scored for each individual item. The threshold for frank psychotic symptoms is defined by operationalized, clear-cut levels of positive symptoms occurring for at least 1 week (see item 9 of the CAARMS-S interview in the Supplementary S3). The Positive and Negative Syndrome Scale (PANSS) (Kay et al., 1987; Peralta and Cuesta, 1994) was administered to explore positive, negative, and general psychopathological symptoms. The PANSS is a 30-item heteroadministered scale. Each item is rated from 1 to 7, with higher ratings indicating increasing levels of symptom severity. It has 3 subscales used to rate positive symptoms (7 items), negative symptoms (7 items), and general psychopathology (16 items). The scores for these scales are obtained by the addition of ratings across component items. Therefore, the potential ranges are 7–49 for the positive and negative scales and 16–112 for the general psychopathology scale. Higher scores indicate higher psychopathology.

*Functioning measures*. The Social and Occupational Functioning Assessment Scale (SOFAS) (Spitzer et al., 2000). This heteroadministered numeric scale evaluates the patient’s social and occupational functioning without taking current symptoms into account. It is a single-item scale, and its score ranges from 1 point (subject with no or extremely limited social performance) to 100 (the best social functioning). Higher scores indicate better functioning.

### 2.3 Procedure

The study was approved by the Ethics Committee of the Catalan Union of Hospitals in accordance with the ethical standards of the 1964 Declaration of Helsinki. The procedures and assessments were described to each patient, who then provided written informed consent (in the case of minors, written informed consent was obtained from their legal guardians).

The CAARMS has been adapted to Spanish using the translation—back translation methodology. Two translations into Spanish of the original tool were made by two bilingual, independent experts in scientific translation, after obtaining permission from the original authors. These two Spanish translations were compared, and discrepancies were resolved by two of the authors.

A bilingual English translator, blinded to the original English scales, back-translated the consensus Spanish version to preserve the reliability of the back-translation. The back-translated version was examined by a staff member of the PACE Clinic in Melbourne who was familiar with the original version of the CAARMS.

Evaluators were blinded to participants’ clinical group assignment, and eligibility was determined by referring clinicians. After eligibility screening, patients were assessed in two sessions: baseline and 6-month follow-up. In each session, one rater administered the CAARMS-S (scored by two raters) and the remaining instruments. The assessments were conducted by specialized personnel, including clinical psychologists and psychiatrists, who underwent a collective training workshop focused on the CAARMS interview to ensure the interrater reliability. Additionally, all evaluators received a 4-h training session on the remaining instruments, administered by a psychologist with extensive experience in the clinical assessment of psychiatric patients. Inter-rater reliability for the PANSS-based UHR and FEP criteria exceeded 0.70.

The overall validation procedure was consistent with the methodological approach employed by Miyakoshi et al. (2009), Braham et al. (2014), and Pelizza et al. (2019) to validate the Japanese, the Arabic and Italian versions, respectively. In addition to their measure, we also assessed changes over time and sensitivity to change.

*Inter-rater reliability*. To analyze interrater reliability, CAARMS-S ratings were made simultaneously by two clinicians, both in the room with the patient together, from mental health centers participating in this study. A total of 7 pairs of professionals were involved in data collection to examine interrater reliability in the UHR group. We also assessed the inter-rater agreement for the CAARMS-S-defined UHR criteria.

*Internal consistency*. To complete the assessment of the reliability of the CAARMS-S, the internal consistency of the instrument was assessed through the correlations between different items of the interview measuring the same psychopathological construct (internal coherence).

*Construct validity*. All the participants were interviewed using CAARMS-S and PANSS. PANSS-defined criteria, based on BPRS/CASH UHR criteria (Yung et al., 2005), were used to determine whether participants met the UHR or FEP criteria. The construct validity of the CAARMS-S was tested comparing the UHR, FEP and control groups in terms of the PANSS positive-, negative-, and general psychopathology-symptoms subscale scores and the CAARMS-S positive-, negative-, general psychopathology symptoms, cognitive-, behavioral, and motor-changes, and emotional disturbances domain scores, which include some Huber’s basic-symptoms (such as, subjective experience of cognitive change, subjective complaints of impaired motor functioning, subjective complaints of impaired bodily sensation, subjective complaints of impaired autonomic functioning, subjective emotional disturbance, avolition/apathy and impaired tolerance to normal stress). We hypothesized that the UHR subjects. Severity scores would be intermediate between those of the FEP group and the control group, with these comparisons inter-groups more pronounced in positive symptoms.

*Concurrent validity*. Concurrent validity was assessed by correlating the positive, negative, and general subscales of the CAARMS-S with the corresponding subscales of the PANSS. Consistent with the Japanese and Arabic validation of the CAARMS, we verified the ability of the CAARMS-S to measure negative symptoms by testing the correlation between the CAARMS Emotional Disturbance and Negative Symptoms subscales scores and the PANSS negative subscale scores.

*Predictive validity*. The CAARMS-S was administered at baseline and at 6-month follow-up to test the predictive validity of the CAARMS-S in the UHR group. The PANSS-defined UHR group was classified as UHR(+), FEP, or UHR(−) according to compliance with the CAARMS-S-defined UHR criteria at baseline. After that, we calculated the rate of transition to psychosis at 6 months. The threshold for frank psychotic symptoms was defined according to CAARMS-S criteria. We predicted that the transition rate at 6 months would be comparable to that in other studies in which putatively effective treatments were provided. The UHR group was recruited from a specific care program for people with an incipient psychotic disorder (PAE-TPI) developed in Catalonia, Spain. Referrals to this program came from health (primary care services, emergency services, acute psychiatric units, or other mental health services), educational, and social services. If referrals come directly from the patient or family member, the PAE-TPI team is responsible for assessing, guiding, and carrying out all necessary actions to assume or redirect the case. All referrals were screened to evaluate the fulfilling UHR criteria generally within 2 weeks. Those who did not meet the CAARMS-S-defined UHR criteria were referred to a mental health service appropriate to their needs if required. Patients who met the CAARMS-S-defined UHR criteria were provided a comprehensive intervention consistent with clinical practice guidelines, such as those of the European Psychiatry Association (Schmidt et al., 2015). In this context, regarding pharmacological interventions, the EPA recommends that, where psychological interventions have proved ineffective, they should be complemented by low-dose second-generation antipsychotics in adult UHR patients with severe and progressive UHR symptomatology to achieve symptomatic stabilization that is required for psychological interventions to be effective. Any long-term antipsychotic treatment with a primarily preventive purpose is not recommended. The participants were usually followed up in accordance with their clinical needs. We calculated the rate of prescription of antipsychotic medication in the CAARMS-S-defined UHR group.

*Changes over time and sensitivity to change*. Differences between scores at baseline and at 6-month follow-up were calculated for the CAARMS-S subscales scores and the PANSS subscales scores. Sensitivity to change was assessed by associations between the CAARMS-S score differences across the positive, negative, and general subscales and PANSS score differences in the same subscales.

### 2.4 Data analysis

Data were analyzed using the Statistical Package for the Social Sciences (SPSS) version 22.0 for Windows (SPSS; Chicago, IL, United States).

Descriptive analyses were conducted using the chi-square test and Fisher’s exact test for categorical variables and Student’s test for continuous variables. Intraclass correlation coefficients (ICCs) were employed to estimate inter-rater reliability, while the kappa coefficient was used to estimate the inter-rater agreement on the diagnosis. According to Fleiss (1981), kappa and ICC values below 0.40 can be interpreted as poor, between 0.41 and 0.75 as fair, and above 0.75 as excellent agreement.

We used Cronbach’s *α* coefficient to assess the internal consistency of CAARMS-S and CAARMS-S subscales at baseline. Cronbach’s α values were considered as follows: 0.60 ≤ α < 0.80 adequate, 0.80 ≤ α < 0.85 good and α ≥ 0.85 excellent (Haertel, 2006).

Between-group comparisons at baseline were examined by using the Kruskal–Wallis test, and post-hoc analyses were performed using the Mann–Whitney U test with Bonferroni correction to control type I error rate. Since we need to make comparisons two by two with three groups (UHR, FEP, and Control groups), the application of the Bonferroni correction will lead us to base our decisions on a significance level of 0.05/3 = 0.017 (Field, 2005).

Spearman’s coefficients were used to determine the correlation between PANSS and CAARMS-S subscales scores. We considered the correlation coefficients as follows: <0.3 = small; 0.3–0.5 = moderate; and ≥0.5 = large (Cohen, 1988).

A descriptive analysis (frequencies and percentages) was carried out to calculate the transition rate of full-blown psychosis at 6 months from baseline in the CAARMS-S-defined UHR group.

The Wilcoxon signed-rank test was used to assess change over time between baseline and at 6-month follow-up for CAARMS-S and PANSS subscales scores. The effect size (ES, Rosenthal correlation coefficient) was also estimated, and its values were considered as follows: <0.3 = small; 0.3–0.5 = moderate; and ≥0.5 = large (Cohen, 1988).

We also demonstrated the CAARMS-S’s sensitivity to change using effect size (ES) and standardized response mean (SRM), from baseline to 6 months of follow-up. The ES was calculated as (M2-M1)/Sb, and the SRM was calculated as (M2-M1)/S∆, where M2 and M1 are the mean 6-month and baseline scores, respectively; Sb is the baseline standard deviation; and S∆ is the standard deviation of the mean change score (Pardasaney et al., 2012). An ES of 0.2 reflects a small change, an ES of 0.5 reflects a moderate change, and an ES of 0.8 reflects a large change (Cohen, 1988). Sensitivity to change was determined by Spearman’s correlation coefficients between SRM of the CAARMS-S scale and SRM of the PANSS scale.

## 3 Results

### 3.1 Sample characteristics

A total of 93 participants (36 UHR, 43 FEP, and 14 controls) were assessed with CAARMS-S. The sociodemographic and clinical data of the UHR and FEP groups are presented in Table 1. The two groups showed no differences regarding sociodemographic variables. The control group was matched to the clinical sample on age, gender, and education. As a baseline, in the UHR group, 16 met the APS criteria, 9 met the APS and GDRS criteria, 8 met the GDRS criteria, and 3 met the BLIPS criteria. DUI in UHR participants, defined as the time from the emergence of the first at-risk symptom to initiation of adequate treatment (i.e., any intervention deemed clinically appropriate, primarily psychosocial, with antipsychotic treatment considered only for severe or progressive symptoms), ranged from 2 to 954 weeks (median = 44 weeks; mean = 146.5 weeks). In this group, the mean age at onset of subthreshold positive psychotic symptoms was 20.1 years (SD = 6.7), a total of 17 individuals (47.2%) received antipsychotic drugs, and 11 individuals (30.6%) had a first-degree family member with a psychotic disorder.

**Table 1:** Sociodemographic and clinical data of the UHR and FEP groups at baseline.

Variables	UHR (*n* = 36)	FEP (*n* = 43)	Statistics
Sociodemographic variables			
Age (years), mean (SD)	22.7 (6.0)	23.6 (6.0)	T-test = 0.503, df = 77, *p* = 0.674
Gender, n (%)			
Females	16 (44.4)	15 (34.9)	χ^2^ = 0.751, df = 1, *p* = 0.386
Males	20 (55.6)	28 (65.1)	
Marital status, n (%)			
Single	34 (94.4)	36 (83.7)	Fisher’s exact test, *p* = 0.170
Other marital status (cohabiting or married or divorce or separated or widow)	2 (5.6)	7 (16.3)	
Educational level, n (%)			
≤Primary	17 (47.2)	26 (60.4)	χ^2^ = 0.072, df = 1, *p* = 0.789
>Primary	19 (42.8)	17 (39.6)	
Current living situation, n (%)			
Living with origen family	23 (63.9)	29 (67.4)	χ^2^ = 0.110, df = 1, *p* = 0.740
Living outside origen family	13 (36.1)	14 (32.6)	
Employment status, n (%)			
Active	3 (8.3)	7 (16.3)	Fisher’s exact test, *p* = 0.332
Non-active	33 (91.7)	36 (83.7)	
Clinical variables			
Diagnosis (ICD-10), n (%)			
Schizophrenia	–	5 (11.6)	
Delusional disorder	–	2 (4.7)	
Brief psychotic disorder	–	6 (14.0)	
Schizoaffective disorder	–	2 (4.7)	
Psychotic disorder NOS	3 (8.3)	22 (51.2)	
Substance-related disorder	1 (2.8)	1 (2.3)	
Bipolar affective disorder	1 (2.8)	3 (7.0)	
Depressive disorder	4 (11.1)	2 (4.7)	
Anxiety disorder	13 (36.1)	–	
Adjustment disorder	3 (8.3)	–	
Personality disorder	5 (13.9)	–	
Developmental disorder	2 (5.6)	–	
Mental disorder NOS	4 (11.1)	–	

UHR, Ultra-High Risk; FEP, First-Episode Psychosis; ICD-10, International Classification of Diseases, Tenth Revision; NOS, not otherwise specified. SD, standard deviation; df, degrees of freedom.

### 3.2 Reliability (inter-rater reliability and internal consistency)

The inter-rater reliability of the CAARMS-S total score was 0.92. Similarly, inter-rater reliability was in the excellent range for all the domains [positive symptoms (0.96), cognitive change attention/concentration (0.80), emotional disturbance (0.84), negative symptoms (0.96), behavioral change (0.88), motor/physical changes (0.93) and general psychopathology (0.93)] and for items of the positive symptoms [unusual thought content (0.89), non-bizarre ideas (0.92), perceptual abnormalities (0.97), and disorganized speech (0.85)]. Cohen’s kappa for agreement on CAARMS-S-defined UHR criteria was 0.97 (*p* < 0.001).

The Cronbach’s *α* coefficient for the CAARMS-S was 0.93, with an α level of 0.81 for the positive symptoms, 0.73 for cognitive change attention/concentration, 0.79 for emotional disturbance, 0.81 for negative symptoms, 0.74 for behavioral change, 0.79 for motor/physical changes, and 0.93 for general psychopathology. All Cronbach’s α values exceeded 0.70, indicating adequate internal consistency.

### 3.3 Construct validity

As hypothesized, UHR scores were intermediate between FEP and controls, with significant differences across subscales (Table 2). In particular, the UHR group’s scores were significantly higher than those of the CG group across all PANSS subscales. Moreover, PANSS positive and general psychopathology subscale scores were significantly higher in FEP than in UHR. Although negative symptoms were more severe in the FEP group than in the UHR group, the difference was not significant (*p* = 0.539). We also compared the UHR, FEP, and CG groups on the CAARMS-S domains. The individuals in the UHR group experienced higher scores in all CAARMS-S domains than those in the CG group, and all differences were significant. FEP patients had higher scores than UHR in the positive symptoms, cognitive change attention, emotional disturbance and negative symptoms domains, and all differences, except in the negative symptom domain (*p* = 0.333), were significant.

**Table 2:** Comparison of mean scores of the PANSS subscales and CAARMS-S domains between three groups.

Variables, mean (SD)	UHR (*n* = 36)	FEP (*n* = 43)	CG (*n* = 14)	Test X^2^	*Post-hoc* test
PANSS					
Positive symptoms	13.3 (4.0)	19.3 (7.0)	8.8 (1.9)	34.6**	FEP > ARMS>CG
Negative symptoms	17.0 (7.1)	18.0 (7.6)	9.8 (1.9)	16.7**	FEP = ARMS>CG
General psychopathology	37.5 (9.4)	42.1 (10.4)	22.4 (2.1)	35.4**	FEP > ARMS>CG
CAARMS-S					
Positive symptoms	10.0 (4.4)	16.9 (3.5)	1.1 (1.8)	59.6**	FEP > ARMS>CG
Unusual thought content	2.6 (1.5)	4.4 (2.0)	0 (0)	41.7**	FEP > ARMS>CG
Non-bizarre ideas	3.2 (1.4)	5.6 (0.9)	0.4 (0.6)	68.5**	FEP > ARMS>CG
Perceptual abnormalities	2.1 (1.8)	4.2 (1.9)	0.5 (1.0)	36.3**	FEP > ARMS>CG
Disorganized speech	2.1 (1.3)	2.8 (1.9)	0.3 (0.7)	22.7**	FEP > ARMS>CG
Cognitive change attention	4.2 (1.8)	6.6 (2.7)	1.1 (1.4)	31.1**	FEP > ARMS>CG
Emotional disturbance	5.2 (3.0)	8.4 (4.1)	1.1 (1.5)	26.2**	FEP > ARMS>CG
Negative symptoms	8.3 (3.0)	9.4 (3.7)	2.6 (2.1)	24.9**	FEP = ARMS>CG
Behavioral change	10.8 (3.3)	–	3.1 (2.3)	26.5**	ARMS>CG
Motor/physical changes	6.2 (3.6)	–	1.2 (1.3)	18.4**	ARMS>CG
General psychopathology	17.1 (6.7)	–	8.9 (3.4)	14.7**	ARMS>CG

UHR, Ultra-High Risk; CAARMS-S, Comprehensive Assessment of At-Risk Mental States-Spanish version; CG, Control Group; FEP, First-Episode Psychosis; PANSS, The Positive and Negative Syndrome Scale. SD, standard deviation. *p < 0.01; **p < 0.001.

### 3.4 Concurrent validity

Three items of the PANSS positive symptoms subscale (specifically, delusion, hallucinatory behavior, and conceptual disorganization) and one item of the PANSS general subscale (specifically, unusual thought content) were correlated with the corresponding items of the CAARMS-S positive symptoms domain (Table 3). All items and domains of the CAARMS-S showed large-to-moderate positive and significant correlations with their corresponding items and subscales of the PANSS, except for the item “unusual thought content” which showed low positive significant and small association.

**Table 3:** Spearman correlations of the CAARMS-S scores with PANSS scores (*n* = 93).

CAARMS-S	PANSS	*r*
Positive symptoms	Positive/general^**1**^ symptoms	0.608**
Unusual thought content	Unusual thought content^1^	0.295**
Non-bizarre ideas	Delusion	0.601**
Perceptual abnormalities	Hallucinatory behavior	0.587**
Disorganized speech	Conceptual disorganization	0.517**
Negative symptoms	Negative symptoms	0.495**
Emotional disturbance	Negative symptoms	0.621**
General psychopathology	General psychopathology	0.577**

^1^Symptom belonging to the PANSS general psychopathology subscale; CAARMS-S, Comprehensive Assessment of At-Risk Mental States- Spanish version; PANSS, The Positive and Negative Syndrome Scale. *p < 0.01; **p < 0.001*p < 0.01; **p < 0.001.

### 3.5 Predictive validity

Of the 36 PANSS-defined UHR participants at baseline, 30 were assessed with CAARMS-S at 6-month follow-up. After this period, 14 participants (46.7%) met UHR criteria, and only one participant (3.3%) had transitioned to full-blown psychosis according to CAARMS-S-defined FEP criteria. A total of 15 participants (50%) were prescribed antipsychotics, 1 FEP, 6 APS, 2 APS-GRDS, and 6 UHR(−).

### 3.6 Changes over time

There were statistically significant changes over time regarding all PANSS and CAARMS-S scores. There was a decrease in the severity of symptoms as revealed by changes in PANSS and CAARMS-S scores over time in all subscales/domains. Effect sizes were large for all scores (Table 4).

**Table 4:** Changes over time in the CAARMS-S domains and PANSS subscales in the UHR group (*n* = 30).

Variables	Baseline		6-months follow-up		Differences over time		
	*Mean*	SD	*Mean*	SD	Test (Z)	*p*	ES (*r*)
PANSS							
Positive symptoms	13.3	4.0	11.1	3.4	−2.6	0.080	0.5
Negative symptoms	17.0	7.1	13.7	5.1	−3.3	0.001	0.6
General psychopathology	37.5	9.4	30.0	7.2	−3.7	<0.001	0.7
CAARMS-S							
Positive symptoms	10.0	4.4	7.8	4.5	−3.5	<0.001	0.6
Cognitive change attention	4.3	1.8	3.0	1.4	−3.4	0.001	0.6
Emotional disturbance	5.2	3.0	4.2	3.2	−3.4	0.001	0.6
Negative symptoms	8.2	3.0	5.8	3.2	−3.5	0.001	0.6
Behavior change	10.8	3.3	7.5	3.8	−4.1	<0.001	0.7
Motor/physical changes	6.2	3.6	4.4	3.4	−3.1	0.002	0.6
General psychopathology	17.1	6.7	12.7	5.3	−3.0	0.003	0.6

CAARMS-S, Comprehensive Assessment of At-Risk Mental States- Spanish version; PANSS, The Positive and Negative Syndrome Scale; UHR, Ultra-High Risk. SD, standard deviation; ES, effect size (Rosenthal correlation coefficient).

### 3.7 Sensitivity to change

The effect size (ES) and standardized response mean (SRM) provided consistent results when assessing the sensitivity to change of the PANSS and CAARMS-S scales from baseline to 6-month follow-up in the UHR group. Regarding positive symptoms, both tools showed a similar degree of change, indicating a moderate response. For negative symptoms, the CAARMS-S interview demonstrated a larger effect size and SRM compared to the PANSS scale (large effect vs. small-to-moderate effect). In terms of general psychopathology, the PANSS scale demonstrated greater sensitivity to change, with both the effect size and SRM indicating a larger effect than the CAARMS-S interview, which showed moderate sensitivity (Table 5). In addition, score differences between baseline and 6-month follow-up were calculated by considering the initial standard deviation in each of the three domains of the CAARMS-S interview and the PANSS scale (positive, negative, and general psychopathology). Table 6 shows that Spearman correlations between changes in PANSS general psychopathology scores and changes in CAARMS-S scores were significant. These coefficients were positive and showed a moderate to high degree of correlation, ranging from 0.42 to 0.58. Regarding positive symptoms, Spearman correlations between changes in CAARMS-S positive scores and changes in PANSS positive scores revealed moderate and statistically significant positive associations. No other significant correlations were found.

**Table 5:** Comparative responsiveness estimates of effect size (ES) and standardized response means (SRM) of the CAARMS-S domains and PANSS subscales in the UHR group (*n* = 30).

Variables	ES (Estimated [95% CI])	SRM (Estimated [95% CI])
PANSS		
Positive symptoms	−0.53 [−0.88 to −0.17]	−0.57 [−0.97 to −0.17]
Negative symptoms	−0.42 [−0.77 to −0.07]	−0.46 [−0.69 to −0.23]
General psychopathology	−0.97 [−1.33 to −0.61]	−0.96 [−1.37 to −0.55]
CAARMS-S		
Positive symptoms	−0.51 [−0.86 to −0.16]	−0.51 [−0.82 to −0.20]
Cognitive change attention	−0.77 [−1.12 to −0.42]	−0.80 [−1.14 to −0.46]
Emotional disturbance	−0.48 [−0.83 to −0.13]	−0.46 [−0.71 to −0.21]
Negative symptoms	−0.74 [−1.09 to −0.39]	−0.74 [−1.07 to −0.41]
Behavior change	−1.10 [−1.45 to −0.75]	−0.96 [−1.26 to −0.66]
Motor/physical changes	−0.50 [−0.85 to −0.15]	−0.51 [−0.79 to −0.23]
General psychopathology	−0.57 [−0.92 to −0.22]	−0.61 [−0.94 to −0.30]

CAARMS-S, Comprehensive Assessment of At-Risk Mental States- Spanish version; PANSS, The Positive and Negative Syndrome Scale; UHR, Ultra-High Risk; CI, Confidence Interval; ES, effect size; SRM, standardized response mean.

**Table 6:** Sensitivity to change of the CAARMS-S for participants UHR (*n* = 30).

Variables	CAARMS-S positive symptoms r (p)	CAARMS-S negative symptoms r (p)	CAARMS-S general psychopathology r (p)
Sensitivity to change (*n* = 30)			
PANSS positive	**0.45 (*p* = 0.012)**	0.03 (*p* = 0.880)	0.16 (*p* = 0.394)
PANSS negative	0.92 (*p* = 0.630)	0.12 (*p* = 0.546)	0.14 (*p* = 0.461)
PANSS general	**0.58 (*p* = 0.001)**	**0.50 (*p* = 0.005)**	**0.42 (*p* = 0.023)**

CAARMS-S, Comprehensive Assessment of At-Risk Mental States- Spanish version; PANSS, The Positive and Negative Syndrome Scale; UHR, Ultra-High Risk. Significant differences (*p* < 0.05) marked in bold.

## 4 Discussion

The main purpose of this study was to analyze the psychometric properties of the Spanish version of the CAARMS in a sample of help-seeking young participants at elevated risk of developing a psychotic episode over a relatively short period of time. Our results showed that CAARMS-S has good psychometric properties and is a reliable and valid instrument for evaluating UHR in the Spanish population. The results obtained are similar to those in the original (Yung et al., 2005) and other validated versions, such as the Italian (Pelizza et al., 2019), Japanese (Miyakoshi et al., 2009), and Arabic (Braham et al., 2014) versions, providing initial evidence of good linguistic and cultural adaptation to our setting. As mentioned in the previous Italian CAARMS validation study (Pelizza et al., 2019), our results further support the cross-cultural generalizability of the UHR concept.

The results obtained in regard to reliability are similar, or even higher, to those obtained with the original version and subsequent validations of the instrument. Domains with higher ICCs in the Spanish version than in other validations included: positive symptoms domain (previous validations: 0.64–0.94; Spanish version: 0.96), negative symptoms domain (the range of values in previous validated versions is 0.59–0.88, however, the value in Spanish version is 0.97), emotional disturbance domain (the range of values in previous validated versions is 0.74–0.83, however, the value in Spanish version is 0.84), cognitive domain (the range of values in previous validated versions is 0.39–0.76, however, the value in Spanish version is 0.80), behavioral change (the range of values in previous validated versions is 0.70–0.84, however, the value in Spanish version is 0.88) and motor/physical changes domain (the range of values in previous validated versions is 0.43–0.89, however, the value in Spanish version is 0.93). In the CAARMS-S, the domain with the lowest ICC value was the cognitive domain. In the remaining validated versions, it is also the domain with the lowest range of values. This domain may be particularly challenging to assess objectively, leading to greater variability in the responses of the raters (Braham et al., 2014; Krebs et al., 2014; Miyakoshi et al., 2009; Pelizza et al., 2019; Yokuşoğlu et al., 2021; Yung et al., 2005).

These findings demonstrate that the instrument can be used clinically to assess the broad symptom spectrum presented by help-seeking youths referred to mental health services, though cognitive symptoms should be supplemented with objective cognitive screening. Regarding the agreement in the diagnosis, higher values have been obtained in the Spanish version than in the other validated versions (Braham et al., 2014; Miyakoshi et al., 2009), although all versions with an excellent agreement. In the same way, the result obtained in the internal consistency coefficient is similar to those of previous versions, although somewhat higher (Braham et al., 2014; Pelizza et al., 2019). The Spanish version of the CAARMS, as well as the Arabic and the Italian versions, had satisfactory uniformity between the different items of the instrument.

The validity of the CAARMS-S was assessed with respect to construct validity, concurrent validity, and predictive validity. Regarding construct validity, all the PANSS symptom subscales scores and the CAARMS-S domains scores showed intermediate scores in the UHR group compared to the other two groups, with the FEP group obtaining higher severity. A similar pattern was observed in previous validated versions, such as the Italian (Pelizza et al., 2019), the Japanese (Miyakoshi et al., 2009) and the Arabic (Braham et al., 2014) versions. The Spanish version showed significant differences between the FEP and UHR groups in positive symptoms and in all subscales/domains except for negative symptoms. The Japanese (Miyakoshi et al., 2009) and the Italian (Pelizza et al., 2019) versions also found similar results in negative symptoms, confirming that these symptoms are already present from previous phases at the onset of full-blown psychosis, but they are not a good element of the CAARMS instrument to discriminate between the PANSS-defined UHR group and the PANSS-defined FEP group. Redefining the CAARMS negative symptoms domain or combining the CAARMS interview with specialized instruments for measuring negative symptoms in UHR individuals, such as the Prodromal Interview of Negative Symptoms (PINS) (Pelletier-Baldelli et al., 2017) could be useful to improve the detection of UHR individuals. In this sense, negative symptoms should be considered to enrich detection algorithms of UHR individuals.

Unlike the Spanish version, in other validated versions of the CAARMS, no significant differences between UHR and FEP groups were found in some positive symptoms and in other domains such as emotional disturbance (Miyakoshi et al., 2009; Pelizza et al., 2019). Besides, in these versions, no significant differences among their groups were found in impaired autonomic functioning (Miyakoshi et al., 2009; Pelizza et al., 2019), impaired bodily sensation (Pelizza et al., 2019), impaired motor functioning, and impaired tolerance to normal stress (Miyakoshi et al., 2009), all of them are Huber’s basic symptoms. In this sense, the Spanish version showed better construct validity than the other validated versions across a wide spectrum of symptoms, indicating that symptoms other than positive symptoms could be used to identify individuals at risk of psychosis. Thus, the CAARMS-S has been able to characterize the risk of psychosis, differentiating it from both full-blown psychosis and non-risk of psychosis.

With regards to concurrent validity, this study showed that the positive symptoms, negative symptoms, emotional disturbance, and general psychopathology domains measured by the CAARMS-S correlated with those assessed by the PANSS. These findings demonstrated that the Spanish version of the CAARMS has good concurrent validity in measuring psychopathology, similar to the Italian (Pelizza et al., 2019), Japanese (Miyakoshi et al., 2009), and Arabic (Braham et al., 2014) versions. Thus, both instruments measure the same construct: psychopathology.

To examine the predictive validity of the CAARMS-S, psychopathology was assessed at 6-month follow-up in the UHR group. Earlier scientific evidence shows that 18% of individuals at risk transition to frank psychotic symptoms within 6 months, which is higher than the 3.3% observed in our study (Fusar-Poli et al., 2012b). However, recent meta-analyses and updated cohort studies indicate that transition rates to psychosis among UHR individuals have progressively declined in comparison with earlier cohorts. Contemporary estimates suggest markedly lower transition risks, particularly in specialized early intervention services, and emphasize that the predictive performance of UHR instruments, including the CAARMS, is best conceptualized as moderate rather than excellent (Fusar-Poli et al., 2020; Hartmann et al., 2016; van Os and Guloksuz, 2017). In this context, the low transition rate observed in our study is consistent with findings from recent early detection programs that provide comprehensive, needs-based interventions.

Different factors could be contributing to obtaining lower transition rates: (a) the PANSS-defined UHR criteria have been used to classify our sample. In this sense, an algorithm for a more imminent risk for psychosis was used without considering the basic symptom criteria, as discussed in the study by Fusar-Poli et al. (2012b), which may have led to the exclusion of individuals with more subtle or long-term risk, thereby reducing the overall observed transition rate; (b) A previous meta-analysis has reported that some interventions (e.g., risperidone plus Cognitive Behavioral Therapy [CBT] interventions) targeted to UHR individuals were associated with a significant reduction in transition rates at 6-month follow-up (Devoe et al., 2020). The UHR group of our study was recruited from a specific care program for people with an incipient psychotic disorder providing evidence-based interventions that are supposed to be effective in reducing the transition rates in UHR individuals (i.e., intensive case management, needs-based interventions, family interventions, CBT, pharmacological treatment, among others, within the framework of assertive community treatment); (c) specific detection strategies developed in this early intervention program allow earlier identification of UHR, reducing the duration of subthreshold symptoms and, consequently, improving prognosis (Nkire et al., 2022; Rapp et al., 2017); (d) these programs can improve adherence to treatment and therefore reduce dropout rates to follow-up.

Importantly, current conceptual frameworks increasingly recognize that the primary clinical utility of instruments such as the CAARMS extends beyond the sole prediction of transition to psychosis. The CAARMS enables a comprehensive and standardized assessment of attenuated psychotic symptoms and associated psychopathology, facilitating risk stratification, longitudinal monitoring, and evaluation of symptom change over time. In modern clinical contexts, effective early interventions may alter illness trajectories and reduce observed transition rates without undermining the validity of the UHR construct or the clinical relevance of the CAARMS.

At 6-month follow-up after the provision of care to patients through PAE-TPI (Barajas et al., 2016), we expected a decrease in psychotic symptoms in both CAARMS-S domains vs. PANSS subscales. There were improvements in all CAARMS-S domains and PANSS subscales, except in the PANSS positive subscale. This may reflect greater sensitivity of the CAARMS positive subscale to change at follow-up compared with the PANSS, indicating improved characterization of UHR and potentially fewer false positives. The latter appears less sensitive to changes in subclinical symptoms and may show greater symptom severity.

Comparing the PANSS subscales and the CAARMS-S domains in terms of sensitivity to change across positive symptoms, negative symptoms, and general psychopathology, the results show that both scales are sensitive to change in positive symptoms, with CAARMS-S showing a stronger response in negative symptoms and PANSS showing a stronger response in general psychopathology. It suggests that the PANSS scale and the CAARMS-S interview can capture changes but in different ways, which may be crucial when selecting the most appropriate instrument to evaluate treatment effects. In this sense, the CAARMS-S interview may be a better measure of the progression of negative symptoms, whereas the PANSS scale is more sensitive to reductions in general symptoms over time.

The significant positive correlations for general psychopathology and positive symptoms suggest that either scale could be effectively used to monitor changes in these domains. However, the lack of significant correlations for negative symptoms might indicate that the CAARMS-S interview, rather than the PANSS scale, would be the instrument of choice if monitoring changes in this domain is specifically required. Thus, the CAARMS-S may be better suited to capturing progression in negative symptoms, particularly in the early stages of psychosis. During this phase, symptoms such as alogia, avolition, and anhedonia are often more subtle and complex to detect. Additionally, individuals exhibiting these early negative symptoms often have reduced insight into their condition, making detection and accurate assessment more challenging. These early symptoms may not match the PANSS negative subscale, which may lack the sensitivity to detect the subtle changes in negative symptoms characteristic of early psychosis. Therefore, the CAARMS-S interview would be more specialized to capture these early negative symptoms, whereas the PANSS scale may be more effective in assessing later stages of psychosis, where symptoms are more consolidated. This finding is relevant, given that negative symptoms are now included in many early intervention protocols as a key criterion for identifying individuals at risk of developing schizophrenia or other psychotic disorders (Tran et al., 2023). Indeed, recent advancements in psychosis risk assessment have incorporated machine learning and predictive models to analyze patterns of negative symptoms alongside other markers such as cognitive impairments and family history. These tools aim to enhance early detection and help healthcare professionals identify individuals at higher risk of developing psychosis (Bjornestad et al., 2022; Dwyer et al., 2022).

Furthermore, the robust psychometric properties established for the CAARMS-S highlight its potential utility beyond direct clinical assessment. As interest grows in leveraging artificial intelligence (AI) and machine learning (ML) for early psychosis detection, validated instruments like the CAARMS-S provide a crucial foundation. The structured, quantifiable data derived from this assessment tool across its various domains (positive, negative, cognitive, emotional, etc.) can serve as valuable feature sets for training and validating predictive algorithms. These AI/ML models could potentially identify complex patterns indicative of risk, enhance predictive accuracy, and ultimately contribute to more sophisticated, data-driven approaches to early intervention (Swinckels et al., 2024).

There were certain limitations in our study. First, the sample size was small, particularly in the control group, which may have limited the statistical power to detect differences in group comparisons; however, it was matched to the UHR and FEP groups on three sociodemographic variables. Second, because participants were recruited from a specific early psychosis program (PAE-TPI), it may be difficult to determine the true capacity of the CAARMS-S to capture transition to psychosis. In addition, because participants were referred for suspected emerging psychosis, they may not be representative of the general outpatient population, which restricts comparability with transition rates from population-based studies. Nonetheless, this approach aligns with the majority of the research on transition to psychosis in help-seeking samples, allowing for meaningful comparisons within this context. Finally, our follow-up was limited to 6 months. Longer-term follow-up is recommended to fully detect all the people who will later develop a psychotic disorder episode (McGorry et al., 2009; Nelson et al., 2013; Schultze-Lutter et al., 2016).

## 5 Conclusion

The CAARMS-S interview has been shown to be a reliable and valid tool for assessing and detecting UHR in a Spanish setting and appears to be helpful in predicting transition to psychosis and capturing changes in psychopathology over time. Taking all the above into account, the CAARMS-S could be used as an assessment tool to identify UHR or FEP patients in the context of early intervention programs for incipient psychotic disorders, for use in research and routine practice. Future research on the CAARMS-S interview could include studying the CAARMS criteria to define a UHR subgroup based on negative and emotional symptoms.

## Data Availability

The raw data supporting the conclusions of this article will be made available by the authors, without undue reservation.
