# Supplementary material for: Detection of psychosis risk: reliability and validity of the Spanish version of the Comprehensive Assessment of At-Risk Mental States interview (CAARMS-S)
Source: Front Psychol. 2026 Apr 13;17:1726125. doi: 10.3389/fpsyg.2026.1726125 (PMC13113797; doi:10.3389/fpsyg.2026.1726125)
Supplement: Supplementary file 1 [file Data_Sheet_1.pdf]

Supplement 1. Comparative table of BPRS/CASH-defined criteria and PANSS-defined FEP criteria

| **BPRS/CASH-defined FEP criteria (Yung et al. 2005)** | **PANSS-defined FEP criteria** |
|---|---|
| **Group Psychotic Disorder** | **Group Psychotic Disorder** |
| Presence of at least one of the following symptoms:<br>- formal thought disorder, as defined by a score of 4 or more on the conceptual disorganization subscale BPRS<br>- a score of 4 or more on the suspiciousness subscale of the BPRS, or it is held with strong conviction, as defined by a score of 3 or more on the CASH rating scale for delusions;<br>- hallucinations as defined by a score of 3 or more on the hallucinations subscale of the BPRS;<br>- delusions as defined by a score of 4 or more on the unusual thought content subscale of the BPRS | Presence of at least one of the following symptoms:<br>- score > or = 4 Conceptual Dizorganization item of PANSS<br>- score > or = 4 on Suspiciousness of PANSS<br>- score > or = 3 on Hallucinatory Behaviour of PANSS<br>- score > or = 4 on Delusions of PANSS |
| Frequency of symptoms: at least several times per week. | = |
| The episode is longer than 1week. | = |
