# Supplementary material for: Detection of psychosis risk: reliability and validity of the Spanish version of the Comprehensive Assessment of At-Risk Mental States interview (CAARMS-S)
Source: Front Psychol. 2026 Apr 13;17:1726125. doi: 10.3389/fpsyg.2026.1726125 (PMC13113797; doi:10.3389/fpsyg.2026.1726125)
Supplement: Supplementary file 2 [file Data_Sheet_2.pdf]

Supplement 2. Comparative table of BPRS/CASH-defined UHR criteria and PANSS-defined UHR criteria

**BPRS/CASH-defined UHR criteria (Yung et al. 2005)**	**PANSS-defined UHR criteria**
**Group 1: attenuated psychotic symptoms**	**Group 1: attenuated psychotic symptoms**
Presence of at least one of the following symptoms:<br><br>Ideas of reference, odd beliefs or magical thinking, perceptual disturbance, paranoid ideation, odd thinking and speech, odd behaviour and appearance (2-3 on unusual thought content subscale, 1-2 on hallucinations subscale, 2-3 on suspiciousness subscale or 1-3 on conceptual disorganization subscale of BPRS.	Presence of at least one of the following symptoms:<br><br>- score = 2 or 3 on Conceptual Dizorganization item of PANSS<br><br>- score = 2 or 3 on Suspiciousness of PANSS<br><br>- score = 2 on Hallucinatory Behaviour of PANSS
Held with a reasonable degree of conviction, as defined by a score of 2 on the CASH rating scale for delusions.	- score = 2 or 3 on Delusions of PANSS
Frequency of symptoms: at least several times per week.	=
The period in which attenuated psychotic symptoms are present is at least 1 week and not longer than 5 years.	=

Supplement 2. Comparative table of BPRS/CASH-defined UHR criteria and PANSS-defined UHR criteria

**BPRS/CASH-defined UHR criteria**<br>**(Yung et al. 2005)**	**PANSS-defined UHR criteria**
**Group 2: BLIPS group**	**Group 2: BLIPS group**
Presence of at least one of the following symptoms:<br><br>Ideas of reference, magical thinking, perceptual disturbance, paranoid ideation, odd thinking and speech (4 + on unusual thought content subscale, 3 + on hallucinations subscale, 4 + on suspiciousness subscale (or it is held strong conviction, as defined by a score of 3 or more on the CASH rating scale for delusions), or 4 + on conceptual disorganization subscale of BPRS)	Presence of at least one of the following symptoms:<br><br>- score > or = 4 Conceptual Dizorganization item of PANSS<br><br>- score > or = 4 on Suspiciousness of PANSS<br><br>- score > or = 3 on Hallucinatory Behaviour of PANSS<br><br>- score > or = 4 on Delusions of PANSS
Duration of episode of less than 1 week	=
Symptoms resolve spontaneously	=
The BLIPS must have occurred within the past year.	=

Supplement 2. Comparative table of BPRS/CASH-defined UHR criteria and PANSS-defined UHR criteria

BPRS/CASH-defined UHR criteria (Yung et al. 2005)	PANSS-defined UHR criteria
**Group 3: Trait and state risk factors**	**Group 3: Trait and state risk factors**
Schizotypal personality disorder (as defined by DSM-IV) or a first degree relative with a DSM-IV psychotic disorder.	Presence of schizotypal personality disorder (assessed across SCID-II) or a first degree relative with a diagnotic of psychotic disorder (defined with a clinical interview).
Significant decrease in mental state or functioning: maintained for at least 1 month and not longer than 5 years (reduction in GAF scale of 30 points from premorbid level.	=
The decrease in functioning occurred within the past year	=
