# Supplementary material for: Detection of psychosis risk: reliability and validity of the Spanish version of the Comprehensive Assessment of At-Risk Mental States interview (CAARMS-S)
Source: Front Psychol. 2026 Apr 13;17:1726125. doi: 10.3389/fpsyg.2026.1726125 (PMC13113797; doi:10.3389/fpsyg.2026.1726125)
Supplement: Supplementary file 3 [file Data_Sheet_3.pdf]

Supplement 3. The Spanish version of the Comprehensive Assessment of At-Risk Mental States interview (CAARMS-S).

CAARMS

COMPREHENSIVE ASSESSMENT OF AT RISK MENTAL STATES

EVALUACIÓN INTEGRAL DE ESTADOS MENTALES DE RIESGO:

CAARMS-S (versión española)

A. Yung, L. Phillips, M.B. Simmons, J. Ward, K. Thompson, P. French, P. McGorry

Nombre: ______________________

ID: ______________________

Evaluador/a: ______________________

Fecha: _____ / _____ / _____

pace

the PACE Clinic

ORYGEN Research Centre

University of Melbourne

Department of Psychiatry

Parkville Victoria, Australia

© 2006 Yung, Phillips, Simmons, Ward, Thompson, French, McGorry

Adaptación experimental – CAARMS-S (2025): A. Barajas y Ll. Lalucat

VISIÓN GENERAL DE LA CAARMS

Objetivos:

- Determinar si un individuo cumple criterios de estado de “Riesgo Extremo” (“Ultra High Risk”).
- Descartar o confirmar criterios de psicosis aguda.
- Trazar el desarrollo psicopatológico y funcional, en el tiempo, en jóvenes con un riesgo extremo (Ultra High Risk) de psicosis.

Estructura de la CAARMS:

- Las puntuaciones se obtienen mediante subescalas que contemplan diferentes áreas psicopatológicas y de funcionamiento. De dichas puntuaciones es posible obtener información referente a los objetivos descritos anteriormente.

Visión General de los Síntomas y el Funcionamiento – Cambio Longitudinal:

- En la primera entrevista (no en las de seguimiento), la CAARMS pretende obtener una visión general de la historia del cambio desde el estado premórbido en el entrevistado. Debe utilizarse toda la información disponible.
- **Registre el momento en que se apreció el primer cambio–** fecha y edad del entrevistado en años:

  Fecha: ………………………………
  Edad: ………………………………

- Apunte los primeros síntomas o signos desde el inicio:
  ……………………………………..……
  ……………………………………..……
  ……………………………………..……
  ……………………………………..……
  ……………………………………..……

- Visión general del curso desde ese momento – trace una línea temporal, por ej.:

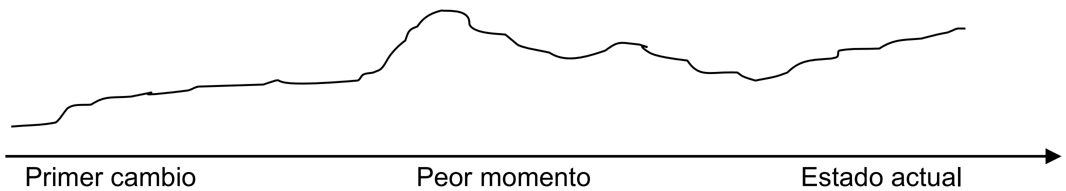

Primer cambio Peor momento Estado actual Tiempo

- Línea temporal actual:

Primer cambio Peor momento Estado actual Tiempo

INDICE

1:	***SÍNTOMAS POSITIVOS***	**página**
1.1	CONTENIDO INUSUAL DEL PENSAMIENTO	1
1.2	IDEAS NO BIZARRAS	3
1.3	ANOMALÍAS PERCEPTIVAS	5
1.4	LENGUAJE DESORGANIZADO	7
2:	***CAMBIO COGNITIVO ATENCIÓN/CONCENTRACIÓN***	
2.1	EXPERIENCIA SUBJETIVA	9
2.2	CAMBIO COGNITIVO OBSERVADO	11
3:	***ALTERACIÓN EMOCIONAL***	
3.1	ALTERACIÓN EMOCIONAL SUBJETIVA	12
3.2	APLANAMIENTO AFECTIVO OBSERVADO	14
3.3	AFECTO INAPROPIADO OBSERVADO	15
4:	***SÍNTOMAS NEGATIVOS***	
4.1	ALOGIA	16
4.2	ABULIA/APATÍA	17
4.3	ANHEDONIA	18
5:	***CAMBIO CONDUCTUAL***	
5.1	AISLAMIENTO SOCIAL	19
5.2	DETERIORO DEL FUNCIONAMIENTO EN EL ROL SOCIAL	20
5.3	CONDUCTA DESORGANIZADA/EXTRAÑA/ESTIGMATIZANTE	21
5.4	CONDUCTA AGRESIVA/PELIGROSA	22
6:	***CAMBIOS MOTORES/FÍSICOS***	
6.1	QUEJAS SUBJETIVAS DE DETERIORO DEL FUNCIONAMIENTO MOTOR	23
6.2	CAMBIOS EN EL FUNCIONAMIENTO MOTOR INFORMADOS U OBSERVADOS	24
6.3	QUEJAS SUBJETIVAS SOBRE EL DETERIORO DE LA SENSACIÓN CORPORAL	25
6.4	QUEJAS SUBJETIVAS DE DETERIORO DEL FUNCIONAMIENTO AUTÓNOMO	26
7:	***PSICOPATOLOGÍA GENERAL***	
7.1	MANIA	27
7.2	DEPRESIÓN	29
7.3	SUICIDALIDAD Y CONDUCTA AUTOLESIVA	31
7.4	OSCILACIONES AFECTIVAS/LABILIDAD	32
7.5	ANSIEDAD	33
7.6	SINTOMAS DE TOC	34
7.7	SINTOMAS DISOCIATIVOS	35
7.8	DISMINUCIÓN DE LA TOLERANCIA AL ESTRÉS HABITUAL	36
8:	***CRITERIOS DE INCLUSIÓN***	37
9:	***UMBRAL DE PSICOSIS***	38

1: SINTOMAS POSITIVOS

1.1 CONTENIDO INUSUAL DEL PENSAMIENTO

Humor Delirante y Perplejidad ('Ideas no Cristalizadas')

- ¿Ha tenido la sensación de que está sucediendo algo extraño que no logra explicarse? ¿Cómo es?
- ¿Se siente confundido? ¿Nota extraños los entornos familiares?
- ¿Siente que ha cambiado de alguna manera?
- ¿Siente que los demás, o el mundo, han cambiado de algún modo?

Ideas de Referencia

- Ideas de Referencia: ¿Ha sentido que las cosas que han estado ocurriendo a su alrededor tienen un significado especial, o que la gente ha estado intentando hacerle llegar mensajes? ¿A qué se parece? ¿Cómo empezó?

Ideas Bizarras ('Ideas Cristalizadas')

- Haber tenido pensamientos, sentimientos, impulsos: ¿Ha sentido que alguien, o algo, fuera de usted, ha estado controlando sus pensamientos, sentimientos, acciones o impulsos?
- Pasividad Somática: ¿Tiene sensaciones corporales extrañas? ¿Sabe qué las causa? ¿Podrían deberse a otras personas o a fuerzas externas?
- Inserción del pensamiento: ¿Ha sentido que ideas o pensamiento, que no son suyos, han sido introducidos en su cabeza? ¿Cómo sabe que no son suyos? ¿De dónde provienen?
- Robo del pensamiento: ¿Ha sentido alguna vez que sus ideas o pensamientos han sido extraídos de su cabeza?¿Cómo ocurre?
- Difusión del pensamiento: ¿Se difunden sus pensamientos de manera que otras personas sepan lo que piensa?
- Lectura del pensamiento: ¿Puede la gente leer su mente?

CONTENIDO INUSUAL DEL PENSAMIENTO
ESCALA DE EVALUACIÓN GLOBAL

0<br>**Nunca, ausente**	1<br>**Dudoso**	2<br>**Leve**	3<br>**Moderado**	4<br>**Moderada-mente grave**	5<br>**Grave**	6<br>**Psicótico y Grave**
No muestra contenido inusual del pensamiento.	Elaboración leve de creencias convenciona-les como podría hacerlo una parte de la población.	Vaga sensación de que algo es diferente, o de que algo no va del todo bien en el mundo, la sensación de que las cosas han cambiado pero sin concretarlo bien.<br>El sujeto no se siente concernido/ preocupado por esta experiencia.	Sentimiento de perplejidad.<br>Mayor sensación de incertidumbre en relación con dichos pensamientos que en 2.	Ideas referenciales de que ciertos acontecimien-tos, objetos o personas tienen un significado particular e inusual.<br>El sujeto siente que sus experiencias pueden provenir de algo externo a él. Creencia no sostenida con convicción, sujeto capaz de cuestionarla.<br>No determina un cambio en la conducta.	Pensamientos inusuales con contenido totalmente original y altamente improbable.<br>El sujeto puede dudar (no los sostiene con convicción delirante), o no los mantiene todo el tiempo.<br>Puede afectar levemente a la conducta.	Pensamientos inusuales con contenido original y altamente improbable sostenido con convicción delirante (sin dudas).<br>Puede tener un marcado impacto en la conducta.

***Fecha de inicio:* ________________ *Fecha de finalización*: ________________**

Frecuencia y duración

0	1	2	3	4	5	6
Ausente	Menos de una vez al mes	De una vez al mes a hasta dos veces por semana – **menos** de una hora por episodio	De una vez al mes hasta dos veces por semana – **más** de una hora por episodio<br>**O**<br>De 3 a 6 veces por semana – **menos** de una hora por episodio	De 3 a 6 veces por semana – **más** de una hora por episodio<br>**O**<br>Diariamente – **menos** de una hora por episodio	Diariamente – **más** de una hora por episodio<br>**O**<br>varias veces al día	Continua-mente

Patrón de Síntomas

0	1	2
No se aprecia relación con el uso de sustancias	Ocurre en relación con el uso de sustancias así como en otras circunstancias	Se aprecia únicamente en relación con el uso de sustancias

Nivel de malestar (En relación a los síntomas)

0 — **100**

Ningún malestar — Malestar extremo

1.2 IDEAS NO BIZARRAS

Ideas No Bizarras ('Ideas Cristalizadas')

- Suspicacia, Ideas de Persecución: ¿Hay alguien que esté molestándolo o tratando de hacerle daño? ¿Siente como si la gente hubiera estado hablando de usted, riéndose, u observándolo? ¿Cómo es?¿Cómo ha llegado a saberlo?
- Ideas de Grandeza: ¿Ha estado sintiendo como si fuera especialmente importante o que posee poderes que otras personas no tienen?
- Ideas Somáticas: ¿Ha tenido la sensación de que algo extraño sucede en su cuerpo que no puede explicarse ? ¿Cómo es? ¿Siente que su cuerpo ha cambiado de alguna manera, o que hay algún problema con su forma?
- Ideas de Culpa: ¿Siente que merece castigo por algo que ha hecho mal?
- Ideas Nihilistas: ¿Ha sentido alguna vez que usted o una parte de usted no existiera o hubiera muerto? ¿Ha sentido alguna vez que el mundo no existiera?
- Ideas de Celos: ¿Es usted una persona celosa? ¿Le preocupan las relaciones que su esposo/a o novio/a mantiene con otras personas?
- Ideas Religiosas: ¿Es usted muy religioso? ¿Ha tenido experiencias religiosas?
- Ideas Erotomaníacas: ¿Hay alguien enamorado de usted? ¿Quién? ¿Cómo lo ha sabido? ¿Corresponde usted?

IDEAS NO BIZARRAS – ESCALA DE EVALUACIÓN GLOBAL

0<br>**Nunca, ausente**	1<br>**Dudoso**	2<br>**Leve**	3<br>**Moderado**	4<br>**Moderada-mente grave**	5<br>**Grave**	6<br>**Psicótico y Grave**
Ausencia de ideas no bizarras.	Cambios sutiles que podrían basarse en la realidad.<br>Ej. marcada conciencia de sí mismo.	Incrementada conciencia de sí mismo.<br>Ej. el sujeto tiene la sensación de que le miran o hablan de él, o sensación de incremento de su importancia.<br>El sujeto es capaz de cuestionarlo.	Pensamientos extraños o inusuales, pero cuyo contenido no es del todo inverosímil, pudiendo haber una evidencia lógica.<br>Mayor evidencia que en el grado 4.<br>El contenido del pensamiento no es original.<br>Ej. celos, leve paranoia.	Creencias claramente idosincráticas que, aunque posibles, sean desarrolladas sin evidencia lógica.<br>Menor evidencia que en el grado 3.<br>Ej. Pensamientos de que otros desean hacerle daño, que pueden ser fácilmente descartados. Pensamientos de poseer poderes especiales, que pueden ser fácilmente descartados.	Pensamientos inusuales (no sostenidos con convicción delirante), o que el sujeto no los mantiene todo el tiempo.<br>Puede producir algún cambio menor de conducta,.	Pensamientos inusuales cuyo contenido es original y altamente improbable, sostenido con convicción delirante (sin ponerlo en duda).<br>Pueden tener un marcado impacto sobre la conducta.

Fecha de inicio: ________________ ***Fecha de finalización***: ________________

Frecuencia y duración

0	1	2	3	4	5	6
Ausente	Menos de una vez al mes	De una vez al mes a hasta dos veces por semana – **menos** de una hora por episodio	De una vez al mes hasta dos veces por semana – **más** de una hora por episodio<br>**O**<br>De 3 a 6 veces por semana – **menos** de una hora por episodio	De 3 a 6 veces por semana – **más** de una hora por episodio<br>**O**<br>Diariamente – **menos** de una hora por episodio	Diariamente – **más** de una hora por episodio<br>**O**<br>varias veces al día	Continua-mente

Patrón de Síntomas

0	1	2
No se aprecia relación con el uso de sustancias	Ocurre en relación con el uso de sustancias así como en otras circunstancias	Se aprecia únicamente en relación con el uso de sustancias

Nivel de malestar (En relación a los síntomas)

0 Ningún malestar — **100** Malestar extremo

1.3 ANOMALÍAS PERCEPTIVAS

Cambios Visuales

- Distorsiones, ilusiones: ¿Ha cambiado la forma en que ve las cosas? ¿Le parecen las cosas de alguna manera diferentes o anormales?¿Han aparecido alteraciones en el color, o en el brillo de los objetos (las cosas aparecen más brillantes o más mates)? ¿Aparecen alteraciones en el tamaño y en la forma de los objetos? ¿Parece que los objetos estén en movimiento?
- Alucinaciones: ¿Tiene visiones, o ve cosas que puede que no estén realmente aquí?¿Ve cosas que otros no pueden o no parecen ver? ¿Qué ve? Cuando ve estas cosas, ¿cuán reales le parecen? ¿Se da cuenta en ese momento de que no son reales o sólo después?

Cambios Auditivos

- Distorsiones, ilusiones: ¿Hay algún cambio en la manera en como suenan las cosas? ¿Los sonidos son de alguna manera diferentes, o anormales? ¿Le parece que la audición es más aguda, o que ha aumentado su sensibilidad? ¿Le parece que su capacidad auditiva ha disminuido, o ha perdido agudeza?
- Alucinaciones: ¿Ha oido cosas que puede que no estén realmente aquí?¿Oye cosas que otras personas no parecen oir (como sonidos o voces)? ¿Qué oye? Cuando oye estas cosas, ¿cuán reales le parecen? ¿Se da cuenta en ese momento de que no son reales o sólo después?

Cambios Olfativos

- Distorsiones, ilusiones: ¿Le parece que su sentido del olfato es diferente, más o menos intenso de lo habitual?
- Alucinaciones: ¿Huele cosas que los demás no perciben? Cuando esto sucede, ¿le parecen reales esos olores? ¿Se da cuenta en ese momento de que no son reales o sólo después?

Cambios Gustativos

- Distorsiones, ilusiones: ¿Le parece que su sentido del gusto es diferente, como más o como menos intenso de lo habitual?
- Alucinaciones: ¿Ha experimentado algún sabor extraño en su boca? Cuando esto sucede, ¿cuán real le parece? ¿Se da cuenta en ese momento de que no son reales o sólo después?

Cambios Táctiles

- Distorsiones, ilusiones, alucinaciones: ¿Ha experimentado alguna vez sensaciones extrañas sobre o bajo su piel? Cuando esto sucede, ¿cuán reales le parecen? ¿Se da cuenta en ese momento de que no son reales o sólo después?

Cambios Somáticos

NOTA: Estas preguntas también se utilizan para puntuar el Deterioro de las Sensaciones Corporales, pág. 26.

- Distorsiones, ilusiones: ¿Ha experimentado sensaciones extrañas en su cuerpo (ej. sentir que partes de su cuerpo han cambiado de alguna manera, o que funcionan de modo distinto). ¿Siente/piensa que hay algún problema en alguna parte o en la totalidad de su cuerpo, por ejemplo que se ve distinto a otros, o que es de alguna manera diferente?¿Cuán real le parece esto?
- Alucinaciones: ¿Ha notado algún cambio en sus sensaciones corporales, como incremento o disminución de intensidad?¿O sensaciones corporales inusuales como presiones, dolores, ardores, entumecimientos, vibraciones?

ANOMALÍAS PERCEPTIVAS – ESCALA DE EVALUACIÓN GLOBAL

0<br>Nunca, ausente	1<br>Dudoso	2<br>Leve	3<br>Moderado	4<br>Moderada-mente grave	5<br>Grave	6<br>Psicótico y Grave
No experimenta anomalías perceptivas.		Percepciones agudizadas o empobrecidas, distorsiones, ilusiones (por ej. luces/ sombras). No especialmente molesto. Experiencias hipnagógicas/ Hipnopómpi-cas.	Más experiencias desconcertan-tes: Distorsiones/ ilusiones más intensas y vívidas, murmullos confusos, etc. El sujeto está inseguro de la naturaleza de dichas experiencias. Capaz de desestimarlas. No inquietantes. Desrealiza-ción/ despersonali-zación.	Experiencias más claras que en 3, tales como ser llamado por su nombre, oír sonar el teléfono, etc., pero fugaces/ transitorias. Es capaz de dar una explicación plausible de estas experiencias. Pueden asociarse con leve malestar.	Verdaderas alucinaciones, por ej., oír voces o conversacio-nes, sentir que algo toca su cuerpo. El sujeto es capaz de cuestionar sus experiencias con esfuerzo. Pueden producir temor o asociarse con algún malestar.	Verdaderas alucinaciones que el sujeto considera reales en el momento de experimentar-las y también después. Pueden causar intenso malestar .

Fecha de inicio: ________________ ***Fecha de finalización***: ________________

Frecuencia y duración

0	1	2	3	4	5	6
Ausente	Menos de una vez al mes	De una vez al mes a hasta dos veces por semana – **menos** de una hora por episodio	De una vez al mes hasta dos veces por semana – **más** de una hora por episodio<br>**O**<br>De 3 a 6 veces por semana – **menos** de una hora por episodio	De 3 a 6 veces por semana – **más** de una hora por episodio<br>**O**<br>Diariamente – **menos** de una hora por episodio	Diariamente – **más** de una hora por episodio<br>**O**<br>varias veces al día	Continua-mente

Patrón de Síntomas

0	1	2
No se aprecia relación con el uso de sustancias	Ocurre en relación con el uso de sustancias así como en otras circunstancias	Se aprecia únicamente en relación con el uso de sustancias

Nivel de malestar (En relación a los síntomas)

0 Ningún malestar **100** Malestar extremo

1.4 LENGUAJE DESORGANIZADO

NOTA: Estas preguntas también se utilizan para puntuar Alogia, p. 16

Cambio subjetivo:

- ¿Ha tenido dificultades en su habla, o habilidad para comunicarse con los demás?
- ¿Ha tenido problemas para encontrar la palabra adecuada en el momento apropiado?
- ¿Utiliza palabras que no vienen al caso, o que son totalmente irrelevantes?
- ¿Se ha encontrado yéndose por la tangente al hablar sin llegar nunca al asunto?¿Es un cambio reciente?
- ¿Se da cuenta de estar hablando de cuestiones irrelevantes, o saliéndose del tema?
- ¿Tienen a veces otras personas dificultad para entender lo que está intentando decir, o para transmitir su mensaje?
- ¿Se encuentra a veces repitiendo las palabras de los demás?
- ¿ Ha tenido que utilizar gestos o mímica para comunicarse debido a problemas para transmitir su mensaje? ¿Cuán desagradable ha sido?
- ¿Le ha causado esto deseos de permanecer en silencio y no decir nada?

Evaluación Objetiva del Lenguaje Desorganizado

- ¿Resulta difícil seguir lo que el sujeto dice debido al uso de palabras incorrectas, a su circunstancialidad y tangencialidad?
- El sujeto es vago, demasiado abstracto o concreto?¿Se pueden sintetizar sus respuestas?
- ¿Suele salirse del tema y perderse al hablar? ¿Parece tener dificultad para encontrar las palabras adecuadas?
- ¿Repite palabras que usted ha utilizado o adopta palabras extrañas (o no palabras) en el curso de una conversación normal?

LENGUAJE DESORGANIZADO – ESCALA DE EVALUACIÓN GLOBAL

0<br>**Nunca, ausente**	1<br>**Dudoso**	2<br>**Leve**	3<br>**Moderado**	4<br>**Moderada-mente grave**	5<br>**Grave**	6<br>**Psicótico**
Discurso lógico, normal, no desorganiza-ción, no problemas de comunicación o de comprensión.		Dificultades subjetivas leves, por ej. problemas para hacerse entender.<br>No perceptible por otros.	Algo vago, alguna evidencia de circunstanciali-dad, o irrelevancia en el discurso.<br>Sensación de no ser entendido.	Clara evidencia de habla y pensa-miento levemente desconec-tados.<br>Asociación de ideas más bien tangencial.<br>Mayor sensación de frustración en la conversa-ción.	Marcada circunstancialidad o tangencialidad en el habla, pero responde estructurándose en la entrevista.<br>Puede tener que recurrir a gestos o mímica para comunicarse.	Ausencia de coheren-cia, habla ininteligi-ble, dificultad significativa para seguir el curso del pensamien-to.<br>Pérdida de asociacio-nes en el habla.

Fecha de inicio: ________________ ***Fecha de finalización***: ________________

Frecuencia y duración

0	1	2	3	4	5	6
Ausente	Menos de una vez al mes	De una vez al mes a hasta dos veces por semana – **menos** de una hora por episodio	De una vez al mes hasta dos veces por semana – **más** de una hora por episodio<br>**O**<br>De 3 a 6 veces por semana – **menos** de una hora por episodio	De 3 a 6 veces por semana – **más** de una hora por episodio<br>**O**<br>Diariamente – **menos** de una hora por episodio	Diariamente – **más** de una hora por episodio<br>**O**<br>varias veces al día	Continua-mente

Patrón de Síntomas

0	1	2
No se aprecia relación con el uso de sustancias	Ocurre en relación con el uso de sustancias así como en otras circunstancias	Se aprecia únicamente en relación con el uso de sustancias

Nivel de malestar (En relación a los síntomas)

0 Ningún malestar — 100 Malestar extremo

2: CAMBIO COGNITIVO – ATENCIÓN/CONCENTRACIÓN

2.1 EXPERIENCIA SUBJETIVA (SÍNTOMA BÁSICO DE HUBER)

Problemas de Atención y Concentración:

- ¿Ha tenido dificultades para concentrarse (dificultades para escuchar a otros, ver la televisión, leer)?
- ¿Le cuesta mucho esfuerzo pensar o concentrarse en algo?

Problemas de Atención Selectiva:

- ¿Le resulta difícil prestar atención a una sola cosa?
- ¿Se distrae con facilidad por otras cosas?
- ¿Se ha sentido abrumado, o confundido por lo que sucede a su alrededor?

Problemas Formales del Pensamiento:

NOTA: Ver también Alogia, p. 16

- ¿Alguna vez le ha parecido que sus pensamientos se han detenido, bloqueado o desaparecido (por ej. “trances”, o “quedarse en blanco”)? ¿Podría describirlo detalladamente?
- ¿Ha experimentado alguna vez pensamientos acelerados, confusos o embrollados?
- ¿Le ha parecido que, además de su pensamiento, parecían detenerse, también, su atención, su audición, su visión, su memoria, su habla o sus movimientos?
- ¿Ha perdido alguna vez su sentido de identidad personal? ¿Cuál cree que ha sido la causa?

Dificultades de Comprensión:

- ¿Tiene dificultad para seguir lo que otros están diciendo?
- ¿Necesita alguna vez que le repitan las frases, en especial si son largas?
- ¿Algunas veces no entiende figuras retóricas o semejantes?
- ¿Supone ésto un cambio para usted, o siempre ha tenido dificultades para ello?
- ¿Ha tenido alguna vez dificultad para captar el tono emocional de las conversaciones (por ej. no reconocer el sarcasmo o la ironía)?
- ¿Le resulta difícil comprender formas no verbales de la comunicación, por ej. gestos? ¿Hasta qué punto le molesta?

Problemas de Memoria:

NOTA: Ver también Síntomas Disociativos, pág. 36

- ¿Ha tenido problemas de memoria?
- ¿Ha sentido alguna vez como si tuviera grandes lagunas en su memoria?
- ¿Están presentes siempre, o aparecen y desaparecen? ¿Ha notado si los problemas de memoria aparecen en momentos de estrés?

CAMBIO COGNITIVO SUBJETIVO – ESCALA DE GRAVEDAD

0<br>**Nunca, Ausente**	1<br>**Dudoso**	2<br>**Leve**	3<br>**Moderado**	4<br>**Moderada-mente grave**	5<br>**Grave**	6<br>**Extremo**
No hay dificultad subjetiva en concentra-ción/<br>atención.	El sujeto es conciente de algunos cambios, pero quizás atribuibles a factores externos.<br>El sujeto tiene dificultad en precisar los cambios.	Leve, pero con problemas definidos, por ej. alguna dificultad para concentrarse al leer o al mirar la TV.<br>Concentrarse requiere un mayor esfuerzo<br>**O**<br>leves dificultades de memoria, pero pasajeras.	Sensación subjetiva de pensamientos embrollados, confusos, acelerados o enlentecidos, dificultad para entender conversacio-nes.<br>Episodios ocasinales de bloqueo del pensamiento<br>**O**<br>problemas de memoria más evidentes pero que no interfieren el funcionamiento cotidiano.	Sensación subjetiva de ser incapaz de pensar adecuada-mente, de confusión, o incapacidad de comprender a otros.<br>Episodios más frecuentes de bloqueo del pensamiento<br>**O**<br>problemas de memoria que dificultan la conversación con frecuentes camvios de asunto.	Marcada pérdida de atención, con sensación, algunas veces, de estar confuso y abrumado, distraido por otras cosas del entorno.<br>Episodios frecuentes de bloqueo del pensamiento<br>**O**<br>los problemas de memoria son apreciados por otros, malestar.	El sujeto manifiesta extrema dificultad para concentrarse en la entrevista.<br>Se debe suspender la entrevista debido a la imposibilidad del paciente para concentrarse o por grave bloqueo del pensamiento<br>**O**<br>graves problemas de memoria.

Fecha de Inicio: ________________ ***Fecha de finalización***: ________________

Frecuencia y duración

0	1	2	3	4	5	6
Ausente	Menos de una vez al mes	De una vez al mes a hasta dos veces por semana – **menos** de una hora por episodio	De una vez al mes hasta dos veces por semana – **más** de una hora por episodio<br>**O**<br>De 3 a 6 veces por semana – **menos** de una hora por episodio	De 3 a 6 veces por semana – **más** de una hora por episodio<br>**O**<br>Diariamente – **menos** de una hora por episodio	Diariamente – **más** de una hora por episodio<br>**O**<br>varias veces al día	Continua-mente

Patrón de Síntomas

0	1	2
No se aprecia relación con el uso de sustancias	Ocurre en relación con el uso de sustancias así como en otras circunstancias	Se aprecia únicamente en relación con el uso de sustancias

2.2 CAMBIO COGNITIVO OBSERVADO

Falta de Atención Observada Durante la Entrevista:

- El sujeto aparece distraido – parece ausente durante la entrevista, no capta el tema durante una discusión, cambia el foco de atención.
- La atención puede desviarse hacia algún sonido de la habitación contigua, hacia objetos de la habitación, hacia la ropa del entrevistador, etc.

Distracción Observada Durante la Evaluación del Estado Mental

- El sujeto muestra una pobre ejecución en las pruebas de funcionamiento intelectual a pesar de disponer de un nivel educativo e intelectual adecuado.
- Esto se evalúa haciendo deletrear al sujeto la palabra "mundo" al revés y haciendo restar series de 7 unidades o series de 3 unidades a la centena, por 5 veces consecutivas.
- **O D N U M**
- **100, 93, 86, 79, 72, 65**
- **100, 97, 94, 91, 88, 85**

CAMBIO COGNITIVO OBSERVADO – ESCALA DE GRAVEDAD

**0**<br>**Nunca, ausente**	**1**<br>**Dudoso**	**2**<br>**Leve**	**3**<br>**Moderado**	**4**<br>**Moderada-mente grave**	**5**<br>**Grave**	**6**<br>**Extremo**
No se observan anomalías.	Alguna distracción dudosa– que podría explicarse por otras causas.	Leves Problemas de concentración.<br>Puede observarse objetivamente pérdida del foco de atención de 1 a 3 veces en la entrevista.<br>No comprende del todo lo que otros están diciendo o el tono emocional en la conversación.	Problemas moderados de concentración durante la entrevista.<br>Leves interrupciones del curso de la entrevista como resultado.	Pobre concentración y atención que afectan significativa-mente la habilidad para ejecutar las tareas.<br>La distracción interfiere claramente el curso de la entrevista.	Dificultades graves de concentración y atención.<br>Extrema dificultad para conducir la entrevista, o seguir un tema debido a la preocupación por estímulos irrelevantes.	Total incapacidad para concentrarse.<br>Imposible conducir la entrevista debido a la preocupación por estímulos irrelevantes.

3: ALTERACIÓN EMOCIONAL

3.1 ALTERACIÓN EMOCIONAL SUBJETIVA (SÍNTOMA BÁSICO DE HUBER)

Empeoramiento del Funcionamiento Emocional:

NOTA: Ver también Anhedonia, pág. 18; Depresión, pág.29

- ¿Ha notado algún cambio en sus sentimientos, o emociones, por ej. sentir que no tiene sentimientos, sentir que sus emociones están vacías, o que sus emociones no son de alguna manera auténticas?
- ¿Ha habido algún cambio en la forma en que maneja sus emociones?
- ¿Puede disfrutar de las cosas, o experimentar placer?
- ¿Piensa que incluso cuando algo triste ocurre, usted ya no es capaz de sentir tristeza?¿ O que cuando algo alegre sucede, no puede sentir alegría?

Cambio en la Afectividad:

Expresión facial:
- ¿Ha notado algún cambio en sus expresiones faciales?
- ¿Le han hecho comentarios sobre su expresión facial diciendo que es neutra, o que es difícil saber lo que está pensando?

Contacto visual:
- ¿Se ha producido algún cambio en el modo en que interactúa con otras personas, por ej. le cuesta mirar a las personas cuando habla con ellas?
- ¿Se lo ha comentado alguien?

Habla:
- ¿Ha apreciado algún cambio en la manera como habla, como si su voz se estuviera volviendo monótona?
- ¿Le ha dicho alguien que tiene una forma monótona de hablar?
- ¿Le parece que lo encuentran aburrido?

Afecto inapropiado:
- ¿Se ha sentido alguna vez distinto interiormente en la manera cómo ve a los demás?
- ¿Es como si su apariencia no estuviera coordinada con sus emociones?¿Ha sonreído o se ha reído mientras hablaba sobre algo triste o nada divertido?

ALTERACIÓN EMOCIONAL SUBJETIVA – ESCALA DE GRAVEDAD

0<br>**Nunca, ausente**	1<br>**Dudoso**	2<br>**Leve**	3<br>**Moderado**	4<br>**Moderada-mente grave**	5<br>**Grave**	6<br>**Extremo**
No hay cambios subjetivos en sentimientos o emociones.		Problemas subjetivos esporádicos leves, pero definidos, por ej. no es capaz de disfrutar de las cosas como antes.<br>Cierta sensación de aplanamiento de las respuestas emocionales.<br>El afecto es inadecuado, pero no sostenido.	Problemas subjetivos más frecuentes o continuos.<br>Cierta sensación de aplanamiento de las respuestas emocionales.<br>Sensación más penetrante afecto inapropiado, pero el sujeto es capaz de controlarlo de algún modo.	El sujeto describe cambios más acusados en las emociones, por ej. no es capaz de expresar o de experimentar los sentimientos como antes.<br>Sensación de distancia-miento cuando está con otros.<br>Afecto inapropiado, más difícil de ocultar a los demás.	El sujeto describe sensación de no tener sentimientos, o sentir sus emociones vacías o no auténticas.<br>Total incapacidad para sentirse triste.<br>Grado grave de distancia-miento de los demás.<br>Afecto inapropiado que interfiere sus relaciones.	El sujeto manifiesta aplanamiento emocional constante<br>O<br>Afecto Inapropiado.

***Fecha de Inicio:* ________________ *Fecha de finalización*: ________________**

Frecuencia y duración

0	1	2	3	4	5	6
Ausente	Menos de una vez al mes	De una vez al mes a hasta dos veces por semana – **menos** de una hora por episodio	De una vez al mes hasta dos veces por semana – **más** de una hora por episodio<br>**O**<br>De 3 a 6 veces por semana – **menos** de una hora por episodio	De 3 a 6 veces por semana – **más** de una hora por episodio<br>**O**<br>Diariamente – **menos** de una hora por episodio	Diariamente – **más** de una hora por episodio<br>**O**<br>varias veces al día	Continua-mente

Patrón de Síntomas

0	1	2
No se aprecia relación con el uso de sustancias	Ocurre en relación con el uso de sustancias así como en otras circunstancias	Se aprecia únicamente en relación con el uso de sustancias

3.2 APLANAMIENTO AFECTIVO OBSERVADO

NOTA: Incorporar información del informante, así como la impresión del entrevistador

- GRADO DE PRESENCIA OBSERVADO DE APLANAMIENTO AFECTIVO. POR EJEMPLO, DISMINUCIÓN DE LA EXPRESIÓN FACIAL, REDUCCIÓN DEL TONO EMOCIONAL DEL HABLA, REDUCCIÓN DE MOVIMIENTOS Y GESTOS EXPRESIVOS.

- El evaluador puede percibir asimismo una disminución de la capacidad para contactar con el sujeto.

____

____

Aplanamiento Afectivo Observado – Escala de Gravedad

0 Nunca, ausente	1 Cuestionable	2 Leve	3 Moderado	4 Moderada-mente grave	5 Grave, no psicótico	6 Extrem/ psicótico
No existen anomalías observadas por el entrevistador u otros.		Se puede observar un grado ligero de disminución del afecto.	Disminución observable del plano emocional. Evitación o fracaso en mostrar sentimientos. Expresión emocional reducida. El entrevistador tiene una sensación de “distancia-miento”, o de contacto empobrecido.	Mayor grado de embotamiento o bloqueo. Disminución acusada de la sensación de contacto observado por el entrevistador. Puede haber sido informada o comentada por los informantes.	Evidencia mínima de expresividad afectiva.	Gran aplanamiento afectivo. No se observa expresión emocional espontánea durante la entrevista. Claramente informado por los informantes.

Fecha de Inicio: ________________ ***Fecha de finalización***: ________________

Frecuencia y duración

0	1	2	3	4	5	6
Ausente	Menos de una vez al mes	De una vez al mes a hasta dos veces por semana – **menos** de una hora por episodio	De una vez al mes hasta dos veces por semana – **más** de una hora por episodio **O** De 3 a 6 veces por semana – **menos** de una hora por episodio	De 3 a 6 veces por semana – **más** de una hora por episodio **O** Diariamente – **menos** de una hora por episodio	Diariamente – **más** de una hora por episodio **O** varias veces al día	Continua-mente

Patrón de Síntomas

0	1	2
No relacionado con el uso de sustancias	Ocurre en relación con el uso de sustancias así como en otras circunstancias	Se aprecia únicamente en relación con el uso de sustancias

3.3 AFECTO INAPROPIADO OBSERVADO

NOTA: Incorporar información del informante, así como la impresión del entrevistador

- También puntuar claramente como afecto inapropiado (afecto claramente discordante con el contenido del habla o de las ideas, por ej. risitas al hablar de algo triste).

____

____

____

AFECTO INAPROPIADO OBSERVADO- ESCALA DE GRAVEDAD

0<br>**Nunca, ausente**	1<br>**Dudoso**	2<br>**Leve**	3<br>**Moderado**	4<br>**Moderada-mente grave**	5<br>**Grave**	6<br>**Extremo**
No existen anomalías observadas por el entrevistador u otros.		Leve afecto inapropiado durante la entrevista, o informado ocasional-mente por otros.<br>El sujeto parece capaz de controlarlo.	Mayor predominio de afectividad inapropiada.<br>No domina la entrevista.<br>El sujeto parece capaz de controlar hasta cierto límite.	Más frecuente-mente informado por otros – produce perturbacio-nes durante la entrevista.	Afecto inapropiado frecuente-mente informado.<br>Interfiere con las relaciones sociales.<br>Interfiere durante la entrevista.	Afecto inapropiado durante la entrevista.<br>Impacta gravemente en la capacidad de conducción de la entrevista.<br>Otros informan que ocurre la mayor parte del tiempo.

Fecha de Inicio: ________________ ***Fecha de finalización***: ________________

Frecuencia y duración

0	1	2	3	4	5	6
Ausente	Menos de una vez al mes	De una vez al mes a hasta dos veces por semana – **menos** de una hora por episodio	De una vez al mes hasta dos veces por semana – **más** de una hora por episodio<br>**O**<br>De 3 a 6 veces por semana – **menos** de una hora por episodio	De 3 a 6 veces por semana – **más** de una hora por episodio<br>**O**<br>Diariamente – **menos** de una hora por episodio	Diariamente – **más** de una hora por episodio<br>**O**<br>varias veces al día	Continua-mente

Patrón de Síntomas

0	1	2
No se aprecia relación con el uso de sustancias	Ocurre en relación con el uso de sustancias así como en otras circunstancias	Se aprecia únicamente en relación con el uso de sustancias

4: SINTOMAS NEGATIVOS

4.1 ALOGIA

NOTA: Véase también Cambio Cognitivo, p9. Lenguaje Desorganizado, p.9

- ¿Ha notado problemas al conversar, por ej. dificultad para encontrar palabras, o bloqueo del pensamiento?
- ¿Son las respuestas del sujeto vagas, o transmiten poca información? ¿Tarda mucho en responder a las preguntas, pero si se le inquiere, parece ser consciente de la pregunta?

ALOGIA – ESCALA DE GRAVEDAD

0<br>**Nunca, ausente**	1<br>**Dudoso**	2<br>**Leve**	3<br>**Moderado**	4<br>**Moderada-mente grave**	5<br>**Grave**	6<br>**Extremo**
No se observan o se informa de cambios en el habla.	El sujeto no está seguro de cambios recientes.<br>Los cambios podrían atribuirse a factores externos, pero el sujeto no está seguro.	Muchos cambios leves en la habilidad para hablar de forma espontánea.<br>El sujeto informa de sentirse "bloqueado" en su pensamiento.<br>Dificultad para encontrar palabras para sus pensamientos.<br>No informado por otros.	Dificultad para expresarse con palabras – encontrar palabras, o mayor frecuencia de episodios de bloqueo del pensamiento.<br>Observable por otros, pero no como dificultad constante.<br>El sujeto responde cuando se le insiste.	Pobreza del habla más marcada, o bloqueo del pensamiento.<br>No interfiere de forma significativa el funciona-miento escolar o laboral.	Incapaz de expresarse de forma adecuada, o grave bloqueo del pensa-miento.<br>Puede experimentar de forma infrecuente periodos de mutismo como resultado de dificultades para encontrar palabras y de expresión.	Marcada pobreza del habla o bloqueo del pensamiento.<br>Dificulta gravemente el curso de la entrevista.<br>El sujeto puede permanecer mudo a ratos.<br>Interfiere significativa-mente con la habilidad para desenvolverse en los ámbitos social, laboral y educativo.

Fecha de Inicio: ________________ ***Fecha de finalización***: ________________

Frecuencia y duración

0	1	2	3	4	5	6
Ausente	Menos de una vez al mes	De una vez al mes a hasta dos veces por semana – **menos** de una hora por episodio	De una vez al mes hasta dos veces por semana – **más** de una hora por episodio<br>**O**<br>De 3 a 6 veces por semana – **menos** de una hora por episodio	De 3 a 6 veces por semana – **más** de una hora por episodio<br>**O**<br>Diariamente – **menos** de una hora por episodio	Diariamente – **más** de una hora por episodio<br>**O**<br>varias veces al día	Continua-mente

Patrón de Síntomas

0	1	2
No relacionado con el uso de sustancias	Ocurre en relación con el uso de sustancias así como en otras circunstancias	Se aprecia únicamente en relación con el uso de sustancias

4.2 ABULIA/APATÍA (SÍNTOMA BÁSICO DE HUBERT)

***Experiencia Subjetiva*:**

- ¿Ha sentido falta de energía-mental y física? ¿Está cansado, o le falta motivación, o iniciativa? ¿Le falta fuerza de voluntad? ¿Falto de fuerza física?
- ¿En qué medida le interfiere esto en actividades como ir a la escuela/trabajo y otras actividades cotidianas? ¿En qué ocupa sus días?

Abulia/Apatía Observada:

NOTA: Ver también Conductas Desorganizada/Extraña/Estigmatizante, pág.21

- ¿Ha señalado el sujeto dificultad para mantener su nivel de compromisos social o laboral/educativo?
- ¿Da la impresión el sujeto de cuidar de sí mismo/a adecuadamente- limpieza, higiene, cuidado general de sí mismo?

Abulia/Apatía – Escala de Gravedad

**0 Nunca, ausente**	**1 Dudoso**	**2 Leve**	**3 Moderado**	**4 Moderadamente grave**	**5 Grave**	**6 Extremo**
No hay cambios observados ni informados de la energía.	El sujeto no está seguro de cambios recientes. Los cambios pueden atribuirse a factores externos, pero no está claro.	Sensación de fatiga, las cosas requieren un esfuerzo. Puede que no inicie actividades como antes. Aun es capaz de llevar a cabo tareas cotidianas. No interfiere con la asistencia escolar o laboral.	Sensación de menor energía, o fuerza de voluntad. Descenso en la asistencia a la escuela/traba-jo, o no realización de las tareas cotidianas como acostumbra. No ocurre a diario y no es informado por otros.	Reducción más marcada de la energía/motivación. Alguna interferencia con el funcionamiento normal, por ej. tarda más en realizar tareas, o el sujeto no se molesta en hacer algunas cosas. Puede faltar a la escuela o al trabajo algunas veces por semana o llegar tarde con frecuencia. Puede ser incapaz de atender su higiene personal.	Reducción diaria de la energía, impuso, fuerza de voluntad, fortaleza física o motivación. Interfiere con el funcionamiento normal, por ej. falta a la escuela o al trabajo la mayoría de los días. Emplea una cantidad significativa de tiempo sin hacer nada. Impacto claro en la higiene personal.	Discapacidad extrema y continuada, por ej. incapaz de realizar tareas normales, recluido en casa sin fuerza de voluntad o volición. Incapaz de asistir a la escuela/trabajo debido a la falta de motivación. Marcado impacto en la higiene personal.

Fecha de Inicio: ________________ ***Fecha de finalización*:** ________________

Frecuencia y duración

0	1	2	3	4	5	6
Ausente	Menos de una vez al mes	De una vez al mes a hasta dos veces por semana – **menos** de una hora por episodio	De una vez al mes hasta dos veces por semana – **más** de una hora por episodio **O** De 3 a 6 veces por semana – **menos** de una hora por episodio	De 3 a 6 veces por semana – **más** de una hora por episodio **O** Diariamente – **menos** de una hora por episodio	Diariamente – **más** de una hora por episodio **O** varias veces al día	Continua-mente

Patrón de Síntomas

0	1	2
No se aprecia relación con el uso de sustancias	Ocurre en relación con el uso de sustancias así como en otras circunstancias	Se aprecia únicamente en relación con el uso de sustancias

4.3 ANHEDONIA

NOTA: Véase también Depresión, pág. 29

- ¿Ha sido capaz de disfrutar de actividades sociales/laborales/de estudio como es habitual? ____________________
- ¿Ha notado una disminución en su nivel de interés por cosas de las que habitualmente disfruta?
- ¿Ha interferido ello en su habilidad para desarrollar actividades, por ej. ir a la escuela/trabajo/participar en acontecimientos? ____________________

ANHEDONIA – ESCALA DE GRAVEDAD

0<br>**Nunca, ausente**	1<br>**Dudoso**	2<br>**Leve**	3<br>**Moderado**	4<br>**Moderada-mente grave**	5<br>**Grave**	6<br>**Extremo**
No se observan ni informan cambios en afecto, habla, nivel de actividad o en concentración.	Leve disminución del interés por los aconteci-mientos, pero puede ser atribuido a causas externas (por ej. no le gusta el tema en la escuela).	Leve disminución del interés por las actividades o en el disfrute de las actividades.<br>No interfiere en la capacidad para realizarlas.	Reducción moderada del interés o del disfrute de actividades como escuela/ trabajo.<br>Puede afectar al rendimiento escuela/ trabajo.	Alguna experiencia común de placer o humor, pero disminuidas en amplitud y calidad. Puede afectar a la asistencia trabajo/ escuela. Otros muestran preocupación por el retraimiento y aislamiento asociados.	Raras veces siente que disfruta o se interesa en las tareas. Algunas veces es capaz de disfrutar de algo, pero brevemente.<br>Baja asistencia a escuela/ trabajo.<br>Muy notorio para otros.	No disfruta ni tiene ningún interés en las tareas.<br>Acusada falta de interés.<br>Aislado y retraido.

Fecha de Inicio: ________________ ***Fecha de finalización***: ________________

Frecuencia y duración

0	1	2	3	4	5	6
Ausente	Menos de una vez al mes	De una vez al mes a hasta dos veces por semana – **menos** de una hora por episodio	De una vez al mes hasta dos veces por semana – **más** de una hora por episodio<br>**O**<br>De 3 a 6 veces por semana – **menos** de una hora por episodio	De 3 a 6 veces por semana – **más** de una hora por episodio<br>**O**<br>Diariamente – **menos** de una hora por episodio	Diariamente – **más** de una hora por episodio<br>**O**<br>varias veces al día	Continua-mente

Patrón de Síntomas

0	1	2
No se aprecia relación con el uso de sustancias	Ocurre en relación con el uso de sustancias así como en otras circunstancias	Se aprecia únicamente en relación con el uso de sustancias

5: CAMBIO CONDUCTUAL

Considerar la información de informantes, así como la información subjetiva

5.1 AISLAMIENTO SOCIAL

- ¿Ha permanecido últimamente en casa más de lo habitual? ¿Ha sido por decisión propia?
- ¿Se ha sentido últimamente incómodo con otras personas?
- ¿Últimamente ha querido estar más tiempo sólo de lo que acostumbra? ¿Ha habido alguna razón para ello? ¿Se lo han comentado otras personas?
- ¿Ha dejado de asistir a actividades sociales/escolares/laborales importantes por este motivo?

***Preguntas para los informantes**:*

- ¿Se ha quedado el sujeto más en casa, quizá solo en su habitación con más frecuencia que antes? Si es así, ¿conoce la razón para ello?
- ¿Ha dejado de asistir a acontecimientos sociales/ trabajo/ escuela por este motivo?
- ¿Parece que prefiera estar más tiempo solo actualmente (más que de costumbre)?

Aislamiento Social- Escala de Gravedad

0<br>**Nunca, ausente**	1<br>**Dudoso**	2<br>**Leve**	3<br>**Moderado**	4<br>**Moderada-mente grave**	5<br>**Grave**	6<br>**Extremo**
No hay cambios en el nivel de actividad social.		El sujeto siente que ya no desea desempeñar sus funciones y roles sociales.<br>Desea estar sólo, pero es capaz de motivarse.	A veces se aísla, pero no marcadamente. Capaz de desempeñar la mayoría de roles y funciones que implican interacción con otros. Puede faltar a algunas actividades sociales.	No tolera estar rodeado de gente durante largos periodos de tiempo.<br>Otros comentan aislamiento social.<br>Puede faltar 2-3 días por semana al colegio/trabajo porque quiere estar solo.	Falta más días de los que asiste al trabajo/ escuela, pasando la mayor parte del día solo.	Mayores periodos aislado de los demás (p. ej. días).

***Fecha de Inicio:** ________________* ***Fecha de finalización:** ________________*

Frecuencia y duración

0	1	2	3	4	5	6
Ausente	Menos de una vez al mes	De una vez al mes a hasta dos veces por semana – **menos** de una hora por episodio	De una vez al mes hasta dos veces por semana – **más** de una hora por episodio<br>**O**<br>De 3 a 6 veces por semana – **menos** de una hora por episodio	De 3 a 6 veces por semana – **más** de una hora por episodio<br>**O**<br>Diariamente – **menos** de una hora por episodio	Diariamente – **más** de una hora por episodio<br>**O**<br>varias veces al día	Continua-mente

Patrón de Síntomas

0	1	2
No se aprecia relación con el uso de sustancias	Ocurre en relación con el uso de sustancias así como en otras circunstancias	Se aprecia únicamente en relación con el uso de sustancias

5.2 DETERIORO DEL FUNCIONAMIENTO EN EL ROL SOCIAL

NOTA: Véase también depresión, pág. 29

- ¿Ha podido ir recientemente al colegio/trabajo como de costumbre?
- ¿Ha empeorado recientemente su rendimiento en el colegio/trabajo?
- ¿Ha estado recientemente menos interesado en su colegio/trabajo?¿Se lo han comentado algunas personas? ¿Hay alguna razón para ello? (Formular adecuadamente la pregunta, por ej. un desempleado)

Preguntas para los informantes:

- ¿Ha apreciado algún algún cambio en la asistencia al colegio/trabajo recientemente?
- ¿Parece el sujeto capaz de realizar tareas habituales como de costumbre?

DETERIORO DEL FUNCIONAMENTO – ESCALA DE GRAVEDAD

0<br>**Nunca, ausente**	1<br>**Dudoso**	2<br>**Leve**	3<br>**Moderado**	4<br>**Moderada-mente grave**	5<br>**Grave**	6<br>**Extremo**
No hay cambios recientes del funciona-miento en el rol social.		El sujeto refiere leve deterioro en la realización de tareas habituales.<br>No apreciado por los informantes.	Tareas habituales realizadas con menor cuidado.<br>Ha faltado ocasional-mente al trabajo/es-cuela. Calificado como leve por los informantes.	Emplea cerca de la mitad del tiempo habitual en las tareas diarias.<br>Otros notan disminución de la calidad en la realización de tareas.	Marcado deterioro del funciona-miento en el rol social.<br>Pasa cerca de la mitad del día en actividad sin propósito.	El sujeto no desarrolla ninguna función social.

Fecha de Inicio: ________________ ***Fecha de finalización***: ________________

Frecuencia y duración

0	1	2	3	4	5	6
Ausente	Menos de una vez al mes	De una vez al mes a hasta dos veces por semana – **menos** de una hora por episodio	De una vez al mes hasta dos veces por semana – **más** de una hora por episodio<br>**O**<br>De 3 a 6 veces por semana – **menos** de una hora por episodio	De 3 a 6 veces por semana – **más** de una hora por episodio<br>**O**<br>Diariamente – **menos** de una hora por episodio	Diariamente – **más** de una hora por episodio<br>**O**<br>varias veces al día	Continua-mente

Patrón de Síntomas

0	1	2
No se aprecia relación con el uso de sustancias	Ocurre en relación con el uso de sustancias así como en otras circunstancias	Se aprecia únicamente en relación con el uso de sustancias

5.3 CONDUCTA DESORGANIZADA/EXTRAÑA/ESTIGMATIZANTE

NOTA: Véase también Abulia, pág. 17; TOC, pág. 34; Aislamiento Social, pág. 19

- ¿Ha habido recientemente alguna cosa en su estilo de vida que otros pueden considerar como inusual, o extraño? (Intente evaluar con precisión conductas peculiares tales como acumular, hablar consigo mismo, movimientos extraños, etc.)
- ¿Puede cuidar de si mismo como acostumbra (bañarse, comer, etc.)? ¿Le han hecho algún comentario al respecto?

Preguntas a los informantes:

- ¿Ha notado recientemente que el sujeto se comporte de forma extraña?
- ¿Ha notado algo extraño en su conducta?¿Se lo han comentado otros?
- ¿Ha notado que esté acumulando objetos, hablando consigo mismo, o moviéndose de forma extraña?

CONDUCTA DESORGANIZADA/EXTRAÑA/ESTIGMATIZANTE – Escala de Gravedad

0<br>**Nunca, ausente**	1<br>**Dudoso**	2<br>**Leve**	3<br>**Moderado**	4<br>**Moderada-mente grave**	5<br>**Grave**	6<br>**Extremo**
No hay cambios en la conducta apreciados por el sujeto, informantes o durante la entrevista.		Cierta disminución, no marcada, en el auto cuidado; aislamiento social.<br>Sujeto capaz de motivarse para rectificar estos cambios.<br>Conducta ligeramente extraña que normalmente no atrae la atención de los demás, o que se produce en privado.	Puede requerir la presión de otros para mantener sus actividades sociales/ laborales o el auto cuidado.<br>Capaz de ser motivado.<br>Conducta extraña ocasional que puede ser notada por otros (por ej. reirse sólo).	Conducta levemente excéntrica claramente apreciable por otros (por ej. hablar consigo mismo/ acumulación de objetos).<br>No continuo.	Conducta claramente bizarra que atrae la atención de otros.<br>Algunas veces da lugar a la intervención de otros.	Auto cuidado muy pobre.<br>Cuadro clínico dominado por conductas excéntricas.<br>Puede producir la intervención de otros.<br>Las conductas extrañas pueden afectar negativamente la salud física.<br>Aislamiento social extremo.

Fecha de Inicio: ________________ ***Fecha de finalización***: ________________

Frecuencia y duración

0	1	2	3	4	5	6
Ausente	Menos de una vez al mes	De una vez al mes a hasta dos veces por semana – **menos** de una hora por episodio	De una vez al mes hasta dos veces por semana – **más** de una hora por episodio<br>**O**<br>De 3 a 6 veces por semana – **menos** de una hora por episodio	De 3 a 6 veces por semana – **más** de una hora por episodio<br>**O**<br>Diariamente – **menos** de una hora por episodio	Diariamente – **más** de una hora por episodio<br>**O**<br>varias veces al día	Continua-mente

Patrón de Síntomas

0	1	2
No se aprecia relación con el uso de sustancias	Ocurre en relación con el uso de sustancias así como en otras circunstancias	Se aprecia únicamente en relación con el uso de sustancias

5.4 CONDUCTA AGRESIVA/PELIGROSA

- ¿Se ha sentido recientemente enfadado o irritable? ¿Ha habido alguna razón para ello? ¿Se ha sentido más irritado de lo habitual por pequeñas cosas? ¿ Ha tenido recientemente más discusiones que habitualmente?¿Ha corrido recientemente más riesgos de lo habitual (por ej. conduciendo)? ¿Le han comentado otros que su conducta se está volviendo arriesgada o poco segura? ¿Ha sentido recientemente el impulso de golpear personas u objetos (más de lo habitual)?
- ¿Se ha sentido tan enfadado con alguien que ha pensado en dañarle, o destruir sus propiedades? ¿Ha llevado a cabo estos pensamientos?

Preguntas a los informantes:

- ¿Ha actuado recientemente el sujeto de forma agresiva o peligrosa? ¿Se ha producido algún episodio reciente de ira/pelea?¿Es esta la conducta habitual del sujeto?¿Han comentado otros algún cambio en su nivel de ira o irritabilidad?¿Ha destruido últimamente propiedades (en asociación con la ira)? ¿Se ha sentido seguro recientemente cuando en compañía del sujeto (por ej. conduciendo, o en otras circunstancias habituales)?

AGRESIVIDAD/CONDUCTA PELIGROSA- ESCALA DE GRAVEDAD

0<br>**Nunca, ausente**	1<br>**Dudoso**	2<br>**Leve**	3<br>**Moderado**	4<br>**Moderada-mente grave**	5<br>**Grave**	6<br>**Extremo**
El sujeto u otros no informan de conducta agresiva o peligrosa.		Ligera irritabilidad, pero no asociada con un incremento de conducta agresiva.<br>El sujeto puede atribuirlo a los aconteci-mientos.	Aumento más marcado en la irritabilidad/ rabia hacia sí mismo/ otros.<br>Puede expresarse verbalmente, o físicamente de forma restringida (por ej. dar puñetazos a la almohada, etc.).<br>Puede ser notado tan sólo por el sujeto.	Marcado incremento de la irritabilidad hacia otros, expresada en una mayor propensión a las discusiones con tendencia a amenaza de agresión física.<br>Notado por otros y por el sujeto.	Conducta agresiva resultante en daños a la propiedad o lesiones a otros.<br>El sujeto informa cierto grado de control sobre la ira.	Peligrosidad en relación con ira, con un alto grado de destructividad, resultando un considerable daño físico a otros o propiedades.<br>Domina el cuadro clínico.<br>Puede atraer la atención de la policía, etc.

Fecha de Inicio: ________________ ***Fecha de finalización***: ________________

Frecuencia y duración

0	1	2	3	4	5	6
Ausente	Menos de una vez al mes	De una vez al mes a hasta dos veces por semana – **menos** de una hora por episodio	De una vez al mes hasta dos veces por semana – **más** de una hora por episodio<br>**O**<br>De 3 a 6 veces por semana – **menos** de una hora por episodio	De 3 a 6 veces por semana – **más** de una hora por episodio<br>**O**<br>Diariamente – **menos** de una hora por episodio	Diariamente – **más** de una hora por episodio<br>**O**<br>varias veces al día	Continua-mente

Patrón de Síntomas

0	1	2
No se aprecia relación con el uso de sustancias	Ocurre en relación con el uso de sustancias así como en otras circunstancias	Se aprecia únicamente en relación con el uso de sustancias

6: CAMBIOS MOTORES/FÍSICOS

6.1 QUEJAS SUBJETIVAS DE DETERIORO DEL FUNCIONAMIETO MOTOR.

(SÍNTOMA BÁSICO DE HUBER)

Movimiento Desorganizado: ______________________

- ¿Ha notado algún cambio en su forma de moverse, por ej. torpeza, falta de coordinación, dificultad para organizar sus actividades o movimientos, pérdida de movimientos espontáneos?
- ¿Ha notado si su habilidad para realizar algunos movimientos se ve afectada por otras cosas?
- ¿Necesita mayor esfuerzo o energía para realizar algunos movimientos?

Manierismos, Posturas Anormales:

- ¿Ha desarrollado algún nuevo movimiento o postura (por ej. un tic nervioso, una forma peculiar de hacer algo, imitar a otros, adoptar ciertas posturas)?¿Qué explicación le encuentra? ______________________

CAMBIO MOTOR SUBJETIVO- ESCALA DE GRAVEDAD

**0**<br>**Nunca, ausente**	**1**<br>**Dudoso**	**2**<br>**Leve**	**3**<br>**Moderado**	**4**<br>**Moderada-mente grave**	**5**<br>**Grave**	**6**<br>**Extremo**
El sujeto no refiere ningún movimiento anormal o dificultad somática.		Solo cambios leves.<br>Sensación de mayor torpeza, más descoordinado de lo habitual, ligera sensación de enlenteci-miento.<br>Muecas ocasionales, o marcha levemente inusual.	Experien-cias señaladas en la columna 1, solo que el sujeto percibe estos cambios de forma más acusada.<br>Refiere capacidad de control sobre ellos.	Cambios como pérdida de coordinación.<br>Los movimientos están afectados por otras cosas.<br>Marcha diferente, nuevas posturas, tics nerviosos o manierismos.<br>Pérdida de habilidades previas.	Experiencias señaladas en la columna 4, pero más estresantes.<br>Puede incluir episodios de mutismo, posturas bizarras, imitación de otros movimientos.	Movimientos claramente distorsionados o idiosincráticos que dominan el cuadro clínico.<br>Manierismos groseros, posturas bizarras.<br>Mudo, o casi mudo, con tan solo muy ocasionales movimientos espontáneos.

Fecha de Inicio: ________________ ***Fecha de finalización***: ________________

Frecuencia y duración

**0**	**1**	**2**	**3**	**4**	**5**	**6**
Ausente	Menos de una vez al mes	De una vez al mes a hasta dos veces por semana – **menos** de una hora por episodio	De una vez al mes hasta dos veces por semana – **más** de una hora por episodio<br>**O**<br>De 3 a 6 veces por semana – **menos** de una hora por episodio	De 3 a 6 veces por semana – **más** de una hora por episodio<br>**O**<br>Diariamente – **menos** de una hora por episodio	Diariamente – **más** de una hora por episodio<br>**O**<br>varias veces al día	Continua-mente

Patrón de Síntomas

**0**	**1**	**2**
No se aprecia relación con el uso de sustancias	Ocurre en relación con el uso de sustancias así como en otras circunstancias	Se aprecia únicamente en relación con el uso de sustancias

6.2 CAMBIOS EN EL FUNCIONAMIENTO MOTOR INFORMADOS U OBSERVADOS:

Movimiento Desorganizado:

- ¿Ha notado algún cambio en la manera en como se mueve, por ej. torpeza, falta de coordinación, dificultades al organizar actividades o movimientos, pérdida de movimientos espontáneos?
- ¿Ha notado si su habilidad para realizar algunos movimientos se ve afectada por otras cosas?
- ¿Requiere más esfuerzo o energía para realizar algunos movimientos?

Manierismos/Posturas Anormales:

- ¿Ha desarrollado nuevos movimientos o posturas (por ej. ha desarrollado un tic nervioso, una forma característica de hacer algo, imitar a otros, adoptar ciertas posturas? ¿Qué explicación le da?

CAMBIO MOTOR OBSERVADO – ESCALA DE GRAVEDAD

0<br>Nunca, ausente	1<br>Dudoso	2<br>Leve	3<br>Moderado	4<br>Moderada-mente grave	5<br>Grave	6<br>Extremo
No se observan ni infoman movimientos anormals o dificultades somáticas.		Otros informan de cambios leves, como por ej. mayor torpeza o descoordi-nación de lo habitual, muecas ocasionales, o marcha levemente inusual.	Experiencias señaladas en la columna 2, pero más marcadas.<br>El sujeto parece tener algún control sobre ellas.	Se informa de que el sujeto tiene dificultades para realizar tareas habituales, como p.ej. conducir.<br>También ha desarrollado nuevos movimientos, p. ej. en la marcha, nuevas posturas/mani erismos.<br>También se puede informar de conductas de imitación.	Se informa de episodios de mutismo y posturas bizarras.<br>No es constante- el sujeto puede detenerlo con ayuda y esfuerzo.	Movimientos claramente distorsionados o idiosincráticos, que dominan el cuadro clínico.<br>Groseros manierismos, posturas bizarras.<br>Mudo, o casi mudo, con tan solo movimientos espontáneos muy ocasionales

6.3 QUEJAS SUBJETIVAS SOBRE DETERIORO DE LA SENSACIÓN CORPORAL
(SÍNTOMA BÁSICO DE HUBER)

NOTA: Ver también pág. 5. Anomalías Perceptivas.

- El sujeto dice que algo va mal en sus sensaciones corporales.
- Ello incluye sensaciones desagradables, aunque cualitativamente normales, como por ej. tirones, dolores, picores, quemazón, entumecimientos, o pueden ser descritas sensaciones cualitativamente anormales, inusuales, o bizarras como "tener cosas" en los ojos, vibraciones, hormigueos.
- ¿Ha sentido alguna vez sensaciones extrañas en su cuerpo (p. ej. sentir que partes de su cuerpo han cambiado de alguna manera, o que funcionan de forma diferente?
- ¿Has sentido/piensa que hay algun problema en alguna parte, o en todo tu cuerpo, por ej. que parece diferente al de los demás o que es de alguna manera diferente?¿Cuán real le parece?

DETERIORO DE LA SENSACIÓN CORPORAL – ESCALA DE GRAVEDAD

0<br>Ausente	1<br>Dudoso	2<br>Leve	3<br>Moderado	4<br>Moderada-mente grave	5<br>Grave	6<br>Extremo
El sujeto informa de que no ha notado ningún cambio en sus sensaciones corporales		El sujeto nota ligeras diferencias ocasionales en sus sensaciones corporales.<br><br>No es constante, capaz de ignorarlo	Informa de cambios más intensos en sus sensaciones corporales.<br><br>Menos capaz de ignorarlos.	Sensaciones corporales bizarras ocasionales.<br><br>Sujeto inseguro de la experiencias.	Informa de mayor número de sensaciones inusuales y bizarras.<br><br>Muy perturbador	Informa de sensaciones corporales en extremo bizarras e inusuales.<br><br>Pueden resultar penosas.

***Fecha de Inicio:* ________________ *Fecha de finalización*: ________________**

Frecuencia y duración

0	1	2	3	4	5	6
Ausente	Menos de una vez al mes	De una vez al mes a hasta dos veces por semana – **menos** de una hora por episodio	De una vez al mes hasta dos veces por semana – **más** de una hora por episodio<br>**O**<br>De 3 a 6 veces por semana – **menos** de una hora por episodio	De 3 a 6 veces por semana – **más** de una hora por episodio<br>**O**<br>Diariamente – **menos** de una hora por episodio	Diariamente – **más** de una hora por episodio<br>**O**<br>varias veces al día	Continua-mente

Patrón de Síntomas

0	1	2
No se aprecia relación con el uso de sustancias	Ocurre en relación con el uso de sustancias así como en otras circunstancias	Se aprecia únicamente en relación con el uso de sustancias

6.4 QUEJAS SUBJETIVAS DE DETERIORO DEL FUNCIONAMIENTO AUTÓNOMO

(SÍNTOMA BÁSICO DE HUBER)

El sujeto puede quejarse de que algo no anda bien en una o más de sus sistemas autónomos siguientes:

- La sensación de que el corazón va muy deprisa o demasiado lento, que respira demsiado deprisa o demasiado profundamente.
- Náuseas.
- Mayor sensibilidad a los cambios de tiempo.
- Necesida de orinar con más frecuencia, estreñimiento.
- Duerme mal, etc.

DETERIORO DE FUNCIONAMIENTO AUTÓNOMO – ESCALA DE GRAVEDAD

0<br>**Ausente**	1<br>**Dudoso**	2<br>**Leve**	3<br>**Moderado**	4<br>**Moderada-mente grave**	5<br>**Grave**	6<br>**Extremo**
No informa.		El sujeto refiere cambios ocasionales en funciones autónomas , por ej. fugaces sensaciones de pánico.<br>No tiene impacto real en las actividades habituales.	Percibe cambios más duraderos, por ej. duerme peor mal varias noches.<br>Interfiere levemente en las actividades habituales.	Se pueden experimentar simultáneamen-te numerosos cambios.<br>Moderada interferencia en las actividades habituales.	Los cambios en las funciones autonómas resultan angustiosos.<br>Ello afecta más marcada-mente las actividades habituales.	El sujeto refiere cambios constantes e intensos en sus funciones autónomas.<br>Muy angustioso.

Fecha de Inicio: ________________ ***Fecha de finalización***: ________________

Frecuencia y duración

0	1	2	3	4	5	6
Ausente	Menos de una vez al mes	De una vez al mes a hasta dos veces por semana – **menos** de una hora por episodio	De una vez al mes hasta dos veces por semana – **más** de una hora por episodio<br>**O**<br>De 3 a 6 veces por semana – **menos** de una hora por episodio	De 3 a 6 veces por semana – **más** de una hora por episodio<br>**O**<br>Diariamente – **menos** de una hora por episodio	Diariamente – **más** de una hora por episodio<br>**O**<br>varias veces al día	Continua-mente

Patrón de Síntomas

0	1	2
No relacionado con el uso de sustancias	Ocurre en relación con el uso de sustancias así como en otras circunstancias	Se aprecia únicamente en relación con el uso de sustancias

7: PSICOPATOLOGÍA GENERAL

7.1 MANÍA

NOTA: Véase también Conducta Agresiva/Peligrosa p. 22

- ¿Describiría su humor reciente como “muy bueno” o “muy alto”?
- Se ha sentido excesivamente alegre y con más energía de lo habitual? ¿Cuánto tiempo ha durado esta sensación?
- ¿Se ha sentido fuera de control durante este tiempo?
- ¿Es esta sensación una respuesta a una sustancia o acontecimiento ocurrido (por ej. finalización de exámenes, nuevo novio/a etc.?
- ¿Ha podido mantenerse despierto haciendo cosas por períodos más largos de lo habitual?
- ¿Ha estado durmiendo menos de lo habitual?
- ¿Se ha encontrado gastando más dinero de lo habitual, o comportándose de manera no normal en usted (por ej. mayor impulso sexual, conducta temeraria, etc.?
- ¿Se ha notado o le han descrito otros hablando más y más rápido de lo habitual?
- ¿Le han comentado que su estado de ánimo, o su energía, parecen más elevados de lo habitual, o fuera de control?
- ¿Se ha sentido recientemente más irritable de lo habitual?¿Ha habido alguna razón para ello?
- ¿Se ha sentido recientemente mejor consigo mismo?
- ¿Se ha sentido de alguna manera especial, o poseedor de poderes o habilidades especiales?

MANÍA – ESCALA DE GRAVEDAD

0<br>Nunca, ausente	1<br>Dudoso	2<br>Leve	3<br>Moderado	4<br>Moderada-mente grave	5<br>Grave	6<br>Extremo
No se observa ni se informa elevación del estado de ánimo.<br>No hay cambios en la autoesti-ma o energía.		Alegre sin mucho motivo.<br>Sensaciones inexplicables de bienestar persistentes o<br>Ligera labilidad del estado de ánimo.<br>Evidencia de exceso de confianza sin base real – dentro de límites normales<br>Y/O<br>Leve irritabilidad.	Refiere excesivas sensaciones de bienestar, o alegría sin una razón subyacente.<br>Algunas veces inapropiado a las circunstancias.<br>Nivel más acusado de excitación.<br>Sentimientos más acusados de autovalía.<br>Ideas sobrevalora-das, no delirante<br>Y/O<br>Irritabilidad moderada.	Sentimientos más persistentes de optimismo, felicidad, o estado de ánimo elevado.<br>Se puede cambiar el estado de ánimo, pero con dificultad.<br>El sujeto es consciente de que sus sentimientos son inapropiados.<br>La conducta puede reflejar su elevado estado de ánimo.<br>Ideas claras de grandiosidad/<br>posesión de poderes especiales, pero no continuas.<br>Irritabilidad más acusada/informa-da por otros.	Estado de ánimo elevado e inapropiado la mayor parte del tiempo.<br>Alguna idea delirante sobre sus poderes/habi lidades.<br>Elevada distraibilidad/ pérdida de asociaciones<br>Entrevista difícil.	El sujeto refiere sentirse exaltado, euforico, marcado incremento de energía, inquietud.<br>La conducta puede ser destructiva-excesivo gasto de dinero/ actividad sexual, etc.<br>Ideas delirantes de grandiosidad/ poder.<br>Fácilmente distráctil, entrevista muy difícil.<br>Sujeto evidentemente irritable.

Fecha de Inicio: ________________ ***Fecha de finalización***: ________________

Frecuencia y duración

0	1	2	3	4	5	6
Ausente	Menos de una vez al mes	De una vez al mes a hasta dos veces por semana – **menos** de una hora por episodio	De una vez al mes hasta dos veces por semana – **más** de una hora por episodio<br>**O**<br>De 3 a 6 veces por semana – **menos** de una hora por episodio	De 3 a 6 veces por semana – **más** de una hora por episodio<br>**O**<br>Diariamente – **menos** de una hora por episodio	Diariamente – **más** de una hora por episodio<br>**O**<br>varias veces al día	Continua-mente

Patrón de Síntomas

0	1	2
No se aprecia relación con el uso de sustancias	Ocurre en relación con el uso de sustancias así como en otras circunstancias	Se aprecia únicamente en relación con el uso de sustancias

7.2 DEPRESIÓN

NOTA: Véase también: Abulia, pág. 17; Anhedonia, pág. 18; Funcionamiento de Rol Social, pág. 20; Ideación Suicida, pág. 31.

- ¿Cómo describiría su estado de ánimo reciente?
- ¿Se ha sentido triste o bajo de ánimos? ¿Con qué frecuencia se ha sentido así?
- De 1 al 10, ¿cuál sería su estado de ánimo medio? ¿Su estado de ánimo más bajo?
- ¿Ha podido disfrutar de actividades, o se ha podido sentir bien consigo mismo?
- ¿Cómo se siente respecto al futuro (evaluar desamparo/desesperanza)?
- ¿Ha disminuído su interés por actividades/eventos?
- ¿Ha sido capaz de realizar, o de iniciar tareas que se había propuesto (evaluar motivación)?
- ¿Cómo ha dormido últimamente (evaluar cambios en el patrón de sueño/insomnio)?
- ¿Cuál ha sido su apetito últimamente? ¿Ha perdido peso?
- ¿Ha ocurrido recientemente alguna cosa que pueda explicar estas sensaciones (fallecimientos / relaciones/ trabajo/ escuela)?

DEPRESIÓN- ESCALA DE GRAVEDAD

0<br>Nunca, ausente	1<br>Dudoso	2<br>Leve	3<br>Moderado	4<br>Moderada-mente grave	5<br>Grave	6<br>Extremo
No refiere estado de ánimo depresivo.<br>No hay signos físicos de depresión.		Algunos sentimientos de tristeza.<br>No domina el cuadro clínico.<br>Capaz de distraerse de pensamientos depresivos.<br>No plantea espontánea-mente asuntos deprimentes.	Evidencia de un estado de ánimo más bajo y sostenido.<br>Mayor dificultad para cambiar el estado de ánimo.<br>El estado de ánimo más bajo puede afectar el nivel de motivación, pero no interfiere significativa-mente el funciona-miento del rol social.<br>Puede estar ligeramente lloroso o con una expresión triste durante la entrevista.	Mayor evidencia observacional de bajo estado de ánimo.<br>Capacidad reducidada para reaccionar frente a eventos placenteros.<br>"Episodios de llanto" más regulares.	Depresión grave – el estado de ánimo no se puede cambiar.<br>No se evidencia componente delirante.<br>Algunas ideas suicidas, pero no llevadas a cabo.<br>Evidentes cambios biológicos consistentes con el bajo estado de ánimo (alteraciones del apetito/sueño)<br>Energía muy baja.	Abatimiento absoluto.<br>El estado de ánimo se acompaña de ideas delirantes, por ej. delirio nihilista.<br>Sentimientos acusados de suicidalidad con conducta asociada.

Fecha de Inicio: ________________ ***Fecha de finalización***: ________________

Frecuencia y duración

0	1	2	3	4	5	6
Ausente	Menos de una vez al mes	De una vez al mes a hasta dos veces por semana – **menos** de una hora por episodio	De una vez al mes hasta dos veces por semana – **más** de una hora por episodio<br>**O**<br>De 3 a 6 veces por semana – **menos** de una hora por episodio	De 3 a 6 veces por semana – **más** de una hora por episodio<br>**O**<br>Diariamente – **menos** de una hora por episodio	Diariamente – **más** de una hora por episodio<br>**O**<br>varias veces al día	Continua-mente

Patrón de Síntomas

0	1	2
No se aprecia relación con el uso de sustancias	Ocurre en relación con el uso de sustancias así como en otras circunstancias	Se aprecia únicamente en relación con el uso de sustancias

7.3 SUICIDALIDAD Y CONDUCTA AUTOLESIVA

- ¿Ha tenido recientemente pensamientos acerca de dañarse, o de matarse? ¿Con qué frecuencia ha pensado estas cosas?
- ¿Ha pensado qué haría para lograrlo?
- ¿Alguna vez ha llevado a cabo dichos pensamientos?¿Qué sucedió?

SUICIDIO – ESCALA DE GRAVEDAD

0<br>**Nunca, ausente**	1<br>**Dudoso**	2<br>**Leve**	3<br>**Moderado**	4<br>**Moderada-mente grave**	5<br>**Grave**	6<br>**Extremo**
No está presente.		Pensamientos ocasionales de estar cansado de vivir.<br>Pensamientos ocasionales de causarse daño.<br>No hay ideación suicida ni planes.	Sentimiento de que estaría mejor muerto.<br>Ideación suicida, con tan solo vaga planificación.<br>Capaz de ser distraído de dichos pensamientos con algún esfuerzo<br>**O**<br>Acciones menores de autolesión (ligeros rasguños,etc).	Ideas de suicidio más frecuentes con un plan asociado.<br>Puede ser más serio si se considera una tentativa con un plan específico<br>**O**<br>Intentos impulsivo utilizando un método no letal, o con conocimiento de la posibilidad de ser hallado.	Expresión clara de quererse matar.<br>**O**<br>Intento potencialmen-te serio o letal con conocimiento de posible rescate.	Plan específico e intento.<br>**O**<br>Intento serio que claramente hubiera podido ser fatal.

Fecha de Inicio: ________________ ***Fecha de finalización***: ________________

Frecuencia y duración

0	1	2	3	4	5	6
Ausente	Menos de una vez al mes	De una vez al mes a hasta dos veces por semana – **menos** de una hora por episodio	De una vez al mes hasta dos veces por semana – **más** de una hora por episodio<br>**O**<br>De 3 a 6 veces por semana – **menos** de una hora por episodio	De 3 a 6 veces por semana – **más** de una hora por episodio<br>**O**<br>Diariamente – **menos** de una hora por episodio	Diariamente – **más** de una hora por episodio<br>**O**<br>varias veces al día	Continua-mente

Patrón de Síntomas

0	1	2
No se aprecia relación con el uso de sustancias	Ocurre en relación con el uso de sustancias así como en otras circunstancias	Se aprecia únicamente en relación con el uso de sustancias

7.4 OSCIACIONES AFECTIVAS/LABILIDAD

- ¿Ha experimentado recientemente cambios anímicos?
- ¿Ha sentido altibajos anímicos sin ninguna razón aparente?
- ¿Puede sentirse alegre un momento y triste al siguiente (o irritable), sin ninguna explicación?
- ¿Con qué frecuencia sucede?
- ¿Ha ocurrido esto en respuesta a drogas, o a eventos que hayan sucedido?¿Se lo han comentado otros?
- ¿Con qué frecuencia ha ocurrido?

OSCILACIONES AFECTIVAS – ESCALA DE GRAVEDAD

0<br>**Nunca, ausente**	1<br>**Dudoso**	2<br>**Leve**	3<br>**Moderado**	4<br>**Moderada-mente grave**	5<br>**Grave**	6<br>**Extremo**
No hay evidencia, ni información de oscilaciones afectivas.		El sujeto refiere que su estado de ánimo cambia más rápidamente de lo habitual. Cambios más acusados en respuesta a sucesos externos. No notado/ informado por otros.	El sujeto refiere cambios más extremos en su estado de ánimo.<br><br>Sensación de que algunas veces el estado de ánimo está fuera de control.	Experiencias más persistentes de oscilaciones afectivas.<br><br>Percibido por otros.<br><br>Causa malestar. Interfiere con las actividades habituales.	Oscilaciones afectivas experimentadas la mayoría de los días.<br><br>Interferencia significativa con las actividades habituales.	El sujeto refiere que sufre oscilaciones afectivas constantes y completa-mente fuera de control.<br><br>Incapaz de mantener un nivel normal de actividad.

Fecha de Inicio: ________________ ***Fecha de finalización***: ________________

Frecuencia y duración

0	1	2	3	4	5	6
Ausente	Menos de una vez al mes	De una vez al mes a hasta dos veces por semana – **menos** de una hora por episodio	De una vez al mes hasta dos veces por semana – **más** de una hora por episodio<br>**O**<br>De 3 a 6 veces por semana – **menos** de una hora por episodio	De 3 a 6 veces por semana – **más** de una hora por episodio<br>**O**<br>Diariamente – **menos** de una hora por episodio	Diariamente – **más** de una hora por episodio<br>**O**<br>varias veces al día	Continua-mente

Patrón de Síntomas

0	1	2
No se aprecia relación con el uso de sustancias	Ocurre en relación con el uso de sustancias así como en otras circunstancias	Se aprecia únicamente en relación con el uso de sustancias

7.5 ANSIEDAD

- ¿Se ha sentido nervioso, o ansioso, recientemente? ¿Ha habido alguna razón para ello? ¿Con qué frecuencia se ha sentido así?
- ¿Cuánto tiempo permanece esta sensación?
- ¿Ha sentido pánico últimamente?
- ¿Ha habido momentos en que ha sentido que le faltaba la respiración, el pulso acelerado, las manos sudorosas, hormigueos en los dedos, sin razón aparente?
- ¿Tiene alguna fobia?¿Le dan miedo los perros, las arañas, los lugares cerrados, las multitudes, etc.?
- ¿Se ha sentido recientemente nervioso en presencia de otros? (distíngase entre ansiedad social y suspicacia).

ANSIEDAD- ESCALA DE GRAVEDAD

**0**<br>**Nunca, ausente**	**1**<br>**Dudoso**	**2**<br>**Leve**	**3**<br>**Moderado**	**4**<br>**Moderada-mente grave**	**5**<br>**Grave**	**6**<br>**Extremo**
No evidencia, ni refiere ansiedad.		Preocupacio-nes menores.<br>Puede distraerse de ellas<br>Y/O<br>Signos físicos leves de ansiedad	Preocupa-ciones moderadas, pero el nivel de ansiedad está dentro del rango apropiado al evento<br>Y/O<br>Signos físicos moderados de ansiedad.	El nivel de ansiedad interfiere ligeramente con las actividades habituales.<br>Alguna preocupación con los desencade-nantes.<br>O<br>Síntomas físicos más acusados.	Preocupacion más acusadas, con miedos, sensación de temor.<br>Y/O<br>Síntomas físicos de ansiedad intrusivos y preocupantes.	Nivel de ansiedad discapacitante, sensación de pánico, aterrorizado.

Fecha de Inicio: ________________ ***Fecha de finalización***: ________________

Frecuencia y duración

**0**	**1**	**2**	**3**	**4**	**5**	**6**
Ausente	Menos de una vez al mes	De una vez al mes a hasta dos veces por semana – **menos** de una hora por episodio	De una vez al mes hasta dos veces por semana – **más** de una hora por episodio<br>**O**<br>De 3 a 6 veces por semana – **menos** de una hora por episodio	De 3 a 6 veces por semana – **más** de una hora por episodio<br>**O**<br>Diariamente – **menos** de una hora por episodio	Diariamente – **más** de una hora por episodio<br>**O**<br>varias veces al día	Continua-mente

Patrón de Síntomas

**0**	**1**	**2**
No se aprecia relación con el uso de sustancias	Ocurre en relación con el uso de sustancias así como en otras circunstancias	Se aprecia únicamente en relación con el uso de sustancias

7.6 SÍNTOMAS DE TOC

- ¿Ha tenido pensamientos penosos o intrusivos, que le dan vueltas en la cabeza sin poderlos detener?
- ¿Posee alguna conducta repetitiva que se siente obligado a realizar?
- ¿Hace alguna cosa para evitar que ocurran "cosas malas" (rituales/ supersticiones, etc.)?
- ¿Necesita tener las cosas de una cierta manera, porque de lo contrario se siente extremadamente ansioso?
- ¿Comprueba repetidamente cosas como los interruptores de la luz/el gas/si los electrodomésticos están desconectados/ las puertas cerradas, etc.?

SÍNTOMAS DE TOC – ESCALA DE GRAVEDAD

0<br>**Nunca, ausente**	1<br>**Dudoso**	2<br>**Leve**	3<br>**Moderado**	4<br>**Moderada-mente grave**	5<br>**Grave**	6<br>**Extremo**
No hay pensamien-tos obsesivos, o rumiaciones.<br>No hay conducta compulsiva.		Refiere alguna rumiación o compulsión, pero que no interfieren con las actividades habituales.<br>No consumen tiempo.<br>Puede distraerse de ellas.	Alguna conducta compulsiva en respuesta a pensamientos obsesivos, pero el sujeto puede controlarlas.<br>Y/O<br>Las compulsiones no interfieren en otras actividades.	Pensamiento obsesivo que distrae. Interfiere en la habilidad para realizar el trabajo/estudio habitual<br>Y/O<br>Las compulsiones no quedan restringidas al hogar o al entorno privado.	Pensamiento obsesivo o compulsiones marcadament e agobiantes<br>Y/O<br>Compulsiones casi constantes – notadas por otros.	Los pensamientos obsesivos poseen cualidad casi delirante.<br>Y/OLas compulsiones interfieren con otras actividades, o amenazan la salud física (por ej. acumulación de basura, excesiva limpieza corporal).

***Fecha de Inicio:* ________________ *Fecha de finalización*: ________________**

Frecuencia y duración

0	1	2	3	4	5	6
Ausente	Menos de una vez al mes	De una vez al mes a hasta dos veces por semana – **menos** de una hora por episodio	De una vez al mes hasta dos veces por semana – **más** de una hora por episodio<br>**O**<br>De 3 a 6 veces por semana – **menos** de una hora por episodio	De 3 a 6 veces por semana – **más** de una hora por episodio<br>**O**<br>Diariamente – **menos** de una hora por episodio	Diariamente – **más** de una hora por episodio<br>**O**<br>varias veces al día	Continua-mente

Patrón de Síntomas

0	1	2
No se aprecia relación con el uso de sustancias	Ocurre en relación con el uso de sustancias así como en otras circunstancias	Se aprecia únicamente en relación con el uso de sustancias

7.7 SÍNTOMAS DISOCIATIVOS

Despersonalización:

¿Se ha experimentado a sí mismo como irreal, como si estuviera fuera de su propio cuerpo?
¿O que alguna parte de su cuerpo no le pertenecía?

Desrealización:

NOTA: Véase también Ideas Nihilistas, pág. 3.
¿Ha tenido la sensación de que las cosas que le rodean son irreales?

Problemas Disociativos de la Memoria:

NOTA: Ver también Cambio Cognitivo, pág. 9.
¿Alguna vez se ha encontrado lejos de su ruta habitual sin recordar como había llegado hasta allí?
¿Estaba entonces sometido a estrés?

SÍNTOMAS DISOCIATIVOS – ESCALA DE GRAVEDAD

0 Nunca, ausente	1 Dudoso	2 Leve	3 Moderado	4 Moderada-mente grave	5 Grave	6 Extremo
No refiere sensaciones de despersonaliza-ción/disociación.		Sensaciones leves de despersonali-zación/desrea-lización. No causa malestar ni distracción.	Experiencias disociativas más acusadas. El sujeto expresa cierta preocupación al respecto, pero no muestra marcada preocupación.	Experiencias disociativas asociadas con elevada preocupación. Malestar debido a estas experiencias.	Malestar como resultado de experiencias disociativas. Interfiere de algún modo las actividades habituales (por ej. tiene que dejar el trabajo/es-cuela/situa-ciones sociales).	Sensaciones de despersonaliza-ción/desrealiza-ción extremadamente molestas. Sentimiento de extremo distanciamiento respecto a los demás. Marcados periodos de tiempo en los que el sujeto no es capaz de describir nada de lo que haya estado haciendo, de dónde haya estado, etc.

Fecha de Inicio: ________________ ***Fecha de finalización***: ________________

Frecuencia y duración

0	1	2	3	4	5	6
Ausente	Menos de una vez al mes	De una vez al mes a hasta dos veces por semana – **menos** de una hora por episodio	De una vez al mes hasta dos veces por semana – **más** de una hora por episodio **O** De 3 a 6 veces por semana – **menos** de una hora por episodio	De 3 a 6 veces por semana – **más** de una hora por episodio **O** Diariamente – **menos** de una hora por episodio	Diariamente – **más** de una hora por episodio **O** varias veces al día	Continua-mente

Patrón de Síntomas

0	1	2
No se aprecia relación con el uso de sustancias	Ocurre en relación con el uso de sustancias así como en otras circunstancias	Se aprecia únicamente en relación con el uso de sustancias

7.8 DISMINUCIÓN DE LA TOLERANCIA AL ESTRÉS HABITUAL
(SÍNTOMA BÁSICO DE HUBER)

- ¿Ha notado algún cambio en la manera de afrontar el estrés cotidiano?
- ¿Te has sentido menos capaz que antes de afrontar, o de tolerar, el estrés cotidiano?
- ¿Cuándo ha estado sometido a los estresores cotidianos, se ha sentido excitado, inseguro, tenso, nervioso o ansioso?
- ¿Ha notado que los estresores cotidianos incrementan otras dificultades que esté experimentando?

DISMINUCIÓN DE LA TOLERANCIA AL ESTRÉS – ESCALA DE GRAVEDAD

**0**<br>**Nunca, ausente**	**1**<br>**Dudoso**	**2**<br>**Leve**	**3**<br>**Moderado**	**4**<br>**Moderada-mente grave**	**5**<br>**Grave**	**6**<br>**Extremo**
No refiere deterioro de la tolerancia al estrés común.		Sensación ligera o poco frecuente de no manejar el estrés tan bien como antes.	Leve sensación de estrés en respuesta a situaciones que habitual-mente el sujeto afrontaría con facilidad.<br><br>Ansiedad leve ante estresores cotidianos, pero aún es capaz de afrontarlos.	Sensación más acusada de gran ansiedad, o tensión ante estresores cotidianos, pero es capaz de realizar tareas habituales.<br>Se siente incapaz de afrontar situaciones más estresantes.<br>Infrecuente-mente puede sentirse ansioso sin motivo alguno.	Sensaciones de gran ansiedad, o tensión ante estresores cotidianos.<br>Algunas veces ansioso sin motivo alguno.	Discapacidad extrema, por ej. ante situaciones triviales, o preocupaciones menores se siente abrumado y presa del pánico.<br>Muy ansioso todo el tiempo, aún sin razón aparente.<br>Incapaz de adaptarse a nuevas situaciones.

Fecha de Inicio: ________________ ***Fecha de finalización***: ________________

Frecuencia y duración

0	1	2	3	4	5	6
Ausente	Menos de una vez al mes	De una vez al mes a hasta dos veces por semana – **menos** de una hora por episodio	De una vez al mes hasta dos veces por semana – **más** de una hora por episodio<br>**O**<br>De 3 a 6 veces por semana – **menos** de una hora por episodio	De 3 a 6 veces por semana – **más** de una hora por episodio<br>**O**<br>Diariamente – **menos** de una hora por episodio	Diariamente – **más** de una hora por episodio<br>**O**<br>varias veces al día	Continua-mente

Patrón de Síntomas

0	1	2
No se aprecia relación con el uso de sustancias	Ocurre en relación con el uso de sustancias así como en otras circunstancias	Se aprecia únicamente en relación con el uso de sustancias

8: CRITERIOS DE INCLUSIÓN

LISTA DE CRITERIOS DE INCLUSIÓN

Grupo 1:Grupo de Vulnerabilidad.

Este criterio identifica a jóvenes en riesgo de psicosis debido a la combinación de un rasgo de factor de riesgo y un deterioro significativo del estado y/o del funcionamiento mental.

	SI	NO
• **Historia familiar de psicosis** en familiares de primer grado **O Trastorno Esquizotípico de la Personalidad** del paciente identificado.	☐	☐
**MAS**<br>• **Disminución del 30%** en la puntuación **EEASL** respecto del nivel premórbido, mantenido durante un mes, y que haya ocurrido en los últimos 12 meses **O puntuación EEASL de 50 o menos** durante los últimos 12 meses o más.	☐	☐
**CUMPLE CRITERIOS DE GRUPO 1 – Grupo de Vulnerabilidad**	☐	☐

Grupo 2: Grupo de Psicosis Atenuada

Este criterio identifica a jóvenes en riesgo de psicosis debida a síndrome psicótico subumbral. Es decir, tienen síntomas que no alcanzan el nivel umbral de psicosis debido a su intensidad subumbral (los síntomas no son lo suficientemente graves) o tienen síntomas psicóticos pero con una frecuencia subumbral (los síntomas no ocurren con la suficiente frecuencia).

	SI	NO
**2a) Intensidad subumbral:**		
• **Puntuación de la Escala de Evaluación Global de 3 a 5** en la subescala *Contenido Inusual del Pensamiento*, **3-5** en la subescala de *Ideación No-Bizarra*, **3-4** en la subescala de *Anomalías Perceptivas* **y/o 4-5** en las subescalas de *Lenguaje Desorganizado* de la CAARMS.	☐	☐
**MAS**<br>• **Puntuación en la Escala de Frecuencia de 3-6** en las subescalas *Contenido Inusual del Pensamiento, Ideación No-Bizarra, Anomalías Perceptivas* **y/o** *Lenguaje Desorganizado de la CAARMS* durante **al menos una semana.**	☐	☐
**2b) Frecuencia subumbral:**		
• **Puntuación de la Escala de Evaluación Global de 6** en las subescalas de *Contenido Inusual del Pensamiento*, de **6** *en Ideación No-Bizarra*, de **5-6** en *Anomalías Perceptivas* **y/o de 6** en *Lenguaje Desorganizado* de la CAARMS.		
**MAS**<br>• **Puntuación en la Escala de Frecuencia de 3** en las subescalas *Contenido Inusual del Pensamiento, Ideación No-Bizarra, Anomalías Perceptivas y/o Lenguaje Desorganizado* de la CAARMS.	☐	☐
**MAS (para ambas categorías)**<br>• **Síntomas presentes durante el último año**	☐	☐
**MAS (para ambas categorías)**		
• **Disminución del 30% en la escala EEASL** respecto al funcionamiento premórbido, mantenido durante un mes, y que haya ocurrido en los últimos 12 meses, **O puntuación EEASL de 50 o menos** durante los últimos 12 meses o más.	☐	☐
**CUMPLE CRITERIOS PARA EL GRUPO 2 – Grupo de Psicosis Atenuada**	☐	☐

Grupo 3: Grupo de Síntomas Psicóticos Breves Limitados Intermitentes (BLIPS):

Este criterio identifica jóvenes en riesgo de psicosis debida a historia reciente de síntomas psicóticos francos, que se resuelven espontáneamente (sin medicación antipsicótica) en una semana.

	SI	NO
• **Puntuación en la Escala de Evaluación Global de 6** en las subescalas *Contenido Inusual del Pensamiento*, de **6** en *Ideación No-Bizarra*, de **5 o 6** en *Anomalías Perceptivas* **y/o 6** en *Lenguaje Desorganizado* de la CAARMS.	☐	☐
**MAS**<br>• **Puntuación en la Escala de Frecuencia de 4-6** en *Contenido Inusual del Pensamiento, Ideación No-Bizarra, Anomalías Perceptivas y/o Lenguaje Desorganizado* de la CAARMS.	☐	☐
**MAS**<br>• **Cada episodio de síntomas está presente durante al menos una semana** y los síntomas remiten de forma espontánea en cada ocasión.	☐	☐
**MAS**<br>• **Los síntomas ocurren durante el último año**	☐	☐
**MAS**		
• **Disminución del 30% en EEASL** respecto al nivel premórbido, mantenido durante un mes, y ocurrido en los últimos 12 meses **O puntuación EEASL de 50 o menos** durante al menos los últimos 12 meses.	☐	☐
**CUMPLE CRITERIOS PARA EL GRUPO 3 – Grupo BLIPS**	☐	☐

9: UMBRAL DE PSICOSIS/UMBRAL DE TRATAMIENTO ANTIPSICÓTICO

	SI	NO
• **Puntuación en la Escala de Severidad de 6** en las subescalas *Contenido Inusual del Pensamiento*, de **6** en *Ideación No-Bizarra*, de **5-6** en *Anomalías Perceptivas* y/o **6** en *Lenguaje Desorganizado* de la CAARMS.	☐	☐
**MAS**<br>• **Puntuación en la Escala de Frecuencia igual o mayor de 4** en las subescalas *Contenido Inusual del Pensamiento, Ideación No-Bizarra, Anomalías Perceptivas y/o Lenguaje Desorganizado.*	☐	☐
**MAS**<br>• Los síntomas han estado presentes **más de una semana**.	☐	☐
**CUMPLE CRITERIOS DE UMBRAL DE PSICOSIS**	☐	☐

ESCALA DE EVALUACIÓN DE LA ACTIVIDAD SOCIAL Y LABORAL (EEASL) [1]

La actividad social y laboral debe considerarse dentro de un espectro continuo que va desde un nivel excelente a un deterioro evidente y completo. Debe incluirse el deterioro debido a impedimentos físicos o trastornos mentales siempre y cuando causa y efecto estén relacionados directamente. No se considerean los efectos derivados de la falta de oportunidades o de otras limitaciones ambientales.

Codificación (Nota: utilice códigos intermedios cuando sea apropiado ej: 45, 68, 72).

PUNTUACIÓN:

100 | 91 — Actividad superior en un amplio abanico de actividades

90 | 81 — Actividad buena y efectiva en todas las áreas

80 | 71 — Ligero deterioro de la actividad social, laboral o escolar(ej: conflictos interpersonales poco frecuentes, retrasos escolares transitorios...)

70 | 61 — Algunas dificultades en la actividad social, laboral, escolar, aunque en líneas generales puede considerarse correcta, el individuo mantiene algunas relaciones interpersonales significativas.

60 | 51 — Moderadas dificultades en la actividad social, laboral y escolar (ej: pocos amigos, conflictos con colegas o colaboradores)

50 | 41 — Seria afectación de la actividad social, laboral o escolar (ej: no tiene amigos, le resulta difícil mantenerse en un empleo...)

40 | 31 — Deterioro importante en diversas áreas, las relaciones laborales, escolares o familiares (ej: un hombre deprimido que evita sus amigos, se muestra negligente y es incapaz de obtener un empleo...)

30 | 21 — Incapaz de activiad en la mayor parte de las áreas (ej: pasa el día en la cama, no tiene trabajo, casa ni amigos...)

20 | 11 — A veces no puede mantener la mínima higiene personal y es incapaz de vivir de forma independiente.

10 | 1 — Incapaz permanentemente de mantener la higiene mínima personal. No puede hacer nada sin perjudicarse a sí mismo o a los demás o sin una dosis considerable de apoyo externo (ej: cuidado, supervisión de una enfermera...)

0 — Información inadecuada

---

[1] **Note:** El grado de funcionamiento psicológico total en una escala de 0-100 fue operativizado por Luborsky en la Health-Sickness Rating Scale (Luborsky L: “Clinicians Judgements of Mental Health” *Archives of General Psychiatry* 7: 401-417, 1962). Spitzer y otros colaboradores desarrollaron una revisión de Health-Sickness Rating Scale conocida como la Global Assessment Scale (GAS) (Endicott J, Spitzer RL, Fleiss JL et al: “The Global Assessment Scale: A Procedure for Measuring Overall Severity of Psychiatric Disturbance” *Archives of General Psychiatry* 33: 766-771, 1976). La escala EEASL se deriva de la GAS y su derarrollo se describe en Goldman HH, Skodol AF, Lave TR: “Revising Axis V for DSM-IV: A Review of Measures of Social Functioning: *American Journal of Psychiatry* 149; 1148-1156, 1992.
